# Targeted demethylation of the CDO1 promoter based on CRISPR system inhibits the malignant potential of breast cancer cells

**DOI:** 10.1002/ctm2.1423

**Published:** 2023-09-22

**Authors:** Jiaojiao Yang, Liyue Sun, Xiao‐Yun Liu, Chan Huang, Junling Peng, Xinxin Zeng, Hailin Zheng, Wenjian Cen, Yu‐Xia Xu, Weijie Zhu, Xiao‐Yan Wu, Dongyi Ling, Lu‐Lu Zhang, Mingbiao Wei, Ye Liu, Deshen Wang, Feng‐Hua Wang, Yu‐Hong Li, Qin Li, Ziming Du

**Affiliations:** ^1^ State Key Laboratory of Oncology in South China Sun Yat‐Sen University Cancer Center Guangzhou Guangdong P. R. China; ^2^ Department of Molecular Diagnostics Sun Yat‐sen University Cancer Center Guangzhou Guangdong P. R. China; ^3^ Second Department of Oncology Guangdong Second Provincial General Hospital Guangzhou Guangdong P. R. China; ^4^ Department of Clinical Laboratory Sun Yat‐Sen University Cancer Center Guangzhou Guangdong P. R. China; ^5^ Department of Medical Oncology Sun Yat‐sen University Cancer Center Guangzhou Guangdong P. R. China; ^6^ Guangdong Provincial Key Laboratory of Malignant Tumor Epigenetics and Gene Regulation Guangdong‐Hong Kong Joint Laboratory for RNA Medicine Sun Yat‐Sen Memorial Hospital Sun Yat‐Sen University Guangzhou Guangdong P. R. China; ^7^ Medical Research Center Sun Yat‐Sen Memorial Hospital Sun Yat‐Sen University Guangzhou Guangdong P. R. China

**Keywords:** Breast cancer, CDO1, epigenetic editing, serum methylation biomarker, targeted demethylation system

## Abstract

**Background:**

Cysteine dioxygenase 1 (CDO1) is frequently methylated, and its expression is decreased in many human cancers including breast cancer (BC). However, the functional and mechanistic aspects of CDO1 inactivation in BC are poorly understood, and the diagnostic significance of serum CDO1 methylation remains unclear.

**Methods:**

We performed bioinformatics analysis of publicly available databases and employed MassARRAY EpiTYPER methylation sequencing technology to identify differentially methylated sites in the CDO1 promoter of BC tissues compared to normal adjacent tissues (NATs). Subsequently, we developed a MethyLight assay using specific primers and probes for these CpG sites to detect the percentage of methylated reference (PMR) of the CDO1 promoter. Furthermore, both LentiCRISPR/dCas9‐Tet1CD‐based CDO1‐targeted demethylation system and CDO1 overexpression strategy were utilized to detect the function and underlying mechanism of CDO1 in BC. Finally, the early diagnostic value of CDO1 as a methylation biomarker in BC serum was evaluated.

**Results:**

CDO1 promoter was hypermethylated in BC tissues, which was related to poor prognosis (*p* < .05). The CRISPR/dCas9‐based targeted demethylation system significantly reduced the PMR of CDO1 promotor and increased CDO1 expression in BC cells. Consequently, this leads to suppression of cell proliferation, migration and invasion. Additionally, we found that CDO1 exerted a tumour suppressor effect by inhibiting the cell cycle, promoting cell apoptosis and ferroptosis. Furthermore, we employed the MethyLight to detect CDO1 PMR in BC serum, and we discovered that serum CDO1 methylation was an effective non‐invasive biomarker for early diagnosis of BC.

**Conclusions:**

CDO1 is hypermethylated and acts as a tumour suppressor gene in BC. Epigenetic editing of abnormal CDO1 methylation could have a crucial role in the clinical treatment and prognosis of BC. Additionally, serum CDO1 methylation holds promise as a valuable biomarker for the early diagnosis and management of BC.

## Graphical abstract

- The targeted demethylation system based on CRISPR/dCas9 confirmed the direct involvement of CDO1 promoter hypermethylation in promoting breast cancer cell proliferation, migration and invasion through the inhibition of CDO1 expression.
- CDO1 functions as a tumour suppressor by impeding cell cycle progression, inducing cell apoptosis and ferroptosis.
- Serum CDO1 promoter methylation serves as a reliable marker for the non‐invasive detection of early‐stage breast cancer.

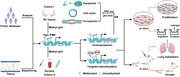

## 1 INTRODUCTION

Breast cancer (BC) has now surpassed lung cancer as the most prevalent type of cancer globally.^1^ However, the mechanism underlying BC's occurrence and development remains unclear. Hypermethylation of the tumour suppressor gene (TSG) is commonly observed in human BC.^2^, ^3^ Cysteine dioxygenase 1 (CDO1) is a non‐haeme iron dioxygenase located on chromosome 5q23.^4^ Its primary function is to catalyse the oxidation of cysteine to cysteine sulfinic acid. This process marks the transition from cysteine catabolism to glutathione synthesis in the presence of molecular oxygen.^5^, ^6^ The hypermethylation of the CDO1 promoter has been linked to unfavourable outcomes in several epithelial solid tumors,^7^, ^8^, ^9^, ^10^, ^11^, ^12^ but its function and underlying mechanism in BC remain poorly understood.

The cytosine bases in the CpG islands (CGIs) of tumour DNA promoters are often methylated to methylcytosine (5mC).^13^ This type of abnormal methylation can disrupt signal transduction in tumour cells and promote tumour activation and progression.^14^ The balance between de novo methyltransferases and ten‐eleven translocation (TET) proteins at promoter and relevant genomic loci allows for quick changes in methylation status, potentially activating or repressing transcription in a locus‐ and cell lineage‐specific manner.^15^ Mammalian TET proteins catalyse the oxidation of 5mC to 5hmC, thereby contributing to DNA demethylation.^16^, ^17^, ^18^, ^19^ TET1, with its Tet catalytic domain (Tet‐CD), plays a crucial role in the demethylation process.^15^ Due to the reversibility of DNA methylation, it has become a hopeful therapeutic target for cancers.^20^, ^21^ The targeted demethylation technology based on the CRISPR/Cas9 system with Tet‐CD was developed to demethylate specifically the target gene without affecting other genes.^19^, ^22^ The CRISPR/Cas9 system utilizes sgRNA to recognize target genomic DNA through base‐complementary pairing, and the nuclease‐deficient Cas9 (dCas9) protein, fused with Tet1 catalytic domain (Tet1CD), achieves targeted demethylation of DNA under the guidance of sgRNA.^19^, ^21^

The 5‐year survival rate of BC with early stages (stages I–II) exceeds 90%, whereas it drops below 25% for stage IV.^23^ Therefore, early detection of BC is crucial for successful treatment and improving survival rates. However, traditional serum tumour antigen biomarkers for BC, such as CEA, CA153, CA19‐9, and CA125, have low sensitivity and specificity, leading to a high rate of missed diagnoses.^24^, ^25^ Therefore, novel non‐invasive biomarkers, particularly for early‐stage BC identification, are needed. Since methylation changes commonly occur in the early stages of cancer, systematic analysis of the methylation profile of serum cell‐free DNA (cfDNA) is being developed for early detection of cancer, monitoring of minimal residual disease, prediction of treatment response and prognosis and tracking tissue origin.^26^, ^27^

**FIGURE 1:**
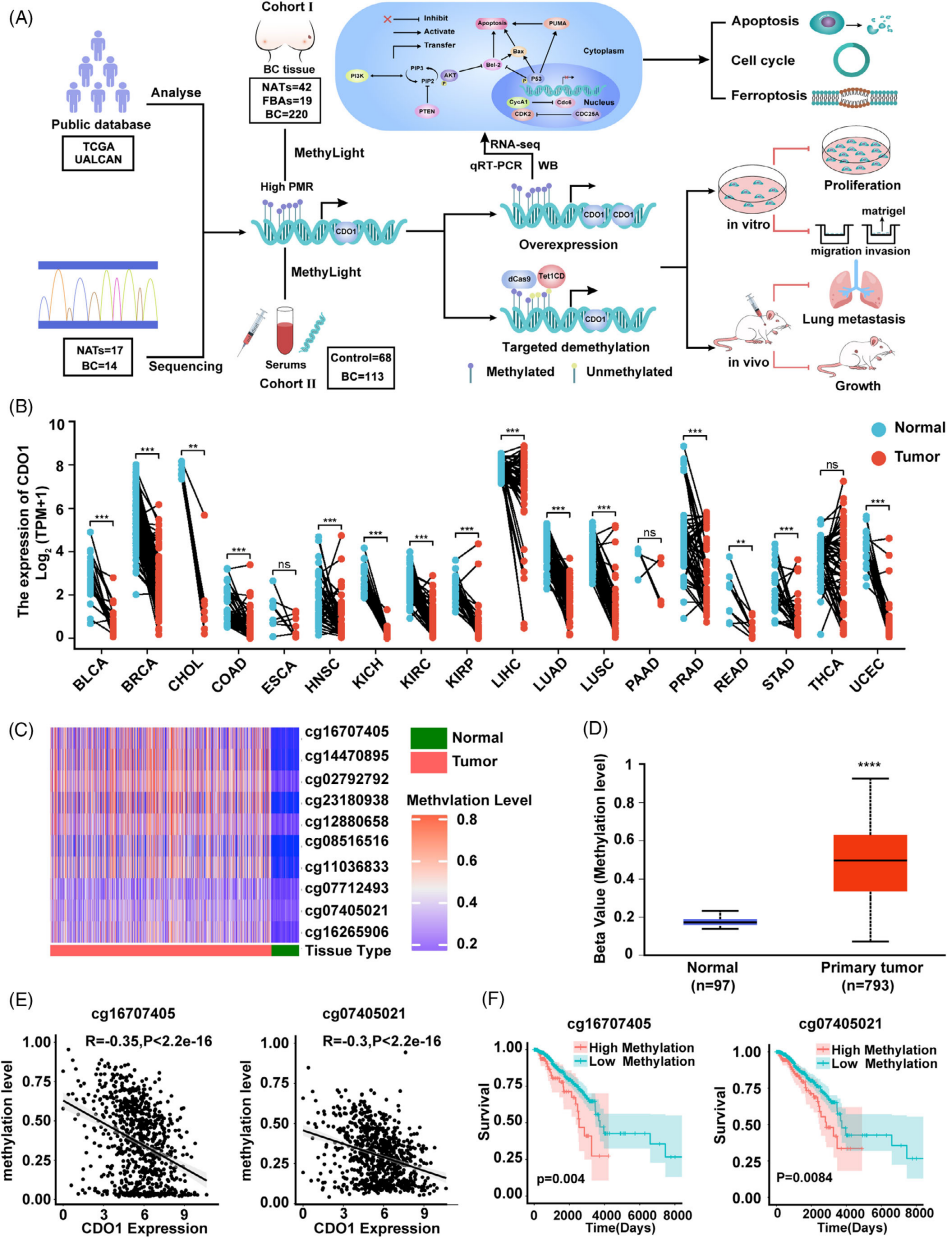
Overview of methylation level, mRNA expression and prognosis of the CDO1 gene in BC tissues. (A) The flow chart of the research in this study. The study followed a sequential approach, involving bioinformatics analysis of publicly available databases and MassARRAY EpiTYPER methylation sequencing to identify differentially methylated sites in the CDO1 promoter region. MethyLight assay was developed to measure the PMR of CDO1 promoter in two cohorts, and the diagnostic value of PMR of serum CDO1 was assessed. The role of CDO1 as a tumour suppressor gene was investigated using a targeted demethylation approach and CDO1 overexpression strategy. Finally, the mechanisms underlying CDO1's anti‐tumour effects, including cell cycle arrest and promotion of cell death, were explored. (B) CDO1 expression was compared between tumour and normal paired samples in pan‐cancer patients. (C) A heatmap illustrated the methylation levels of specific CpG sites within the CDO1 promoter in breast cancer tissues from the TCGA database. (D) The methylation levels of the CDO1 gene were depicted using a boxplot, comparing breast cancer tissues and normal adjacent tissues from the UALCAN database. The beta value cut‐off indicated hypermethylation (.7– .5) or hypomethylation (.3–.25). (E) Pearson correlation analysis was conducted to assess the relationship between the methylation of specific CpG sites (cg16707405 and cg07405021) and gene expression. Statistical significance was indicated by *p* < .05. (F) Kaplan–Meier curves were generated to evaluate the prognosis of breast cancer patients in the hypermethylated and hypomethylated CDO1 groups at cg16707405 and cg07405021 sites (*p* = .004 and .0084, respectively). The statistical analysis employed the log‐rank test. Data were presented as mean ± SD. The notation used is ns, non‐significant; *p < .05, **p < .01, ***p < .001, ****p < .0001.

Considering these findings and aiming to elucidate the function, underlying mechanism and early diagnostic value of CDO1 in BC, we conducted bioinformatics analysis of publicly available databases and MassARRAY EpiTYPER methylation sequencing to identify differentially methylated sites in the CDO1 promoter region between BC tissues and normal adjacent tissues (NATs). The MethyLight assay was then developed with specific primers and probes designed for those CpG sites to detect the percent of methylated reference (PMR) of CDO1 promoter in two cohorts: cohort I of tissue and cohort II of serum, and the early diagnostic value of serum CDO1 PMR were then evaluated. Additionally, both lentiCRISPR/dCas9‐Tet1CD‐sgRNA‐based CDO1‐targeted demethylation and CDO1 overexpression strategy were employed to investigate the role of CDO1 as a TSG. Furthermore, we explored the potential mechanism of the CDO1 anti‐tumour effect and found that CDO1 overexpression arrested cell cycle progression and promoted cell apoptosis and ferroptosis in BC cells (Figure 1A).

## 2 MATERIALS AND METHODS

### 2.1 Public data sources and bioinformatics analysis

The pan‐cancer sequencing expression data were retrieved from *The Cancer Genome Atlas (TCGA)*. Pan‐cancer included normal tissues (n = 1414) and cancer tissues (n = 10,494), involving paired samples (n = 687) and unpaired samples (n = 10,534). The CDO1 promoter methylation data of BC were obtained from the TCGA IlluminaHuman450K methylation database, which comprises a total of 865 samples, consisting of 781 BC samples and 84 NATs. We also downloaded CDO1 methylation data from the UALCAN database including 793 BC samples and 97 normal samples. The beta value represents the degree of DNA methylation, ranging from 0 (unmethylated) to 1 (fully methylated). We calculated the correlation between the methylation level and gene expression using Spearman method (*p* < .05). The data obtained from TCGA and UALCAN are publicly available without permission from the ethics committee.

### 2.2 Patient samples

The cohort I of tissue samples, including 220 cases of BC tissues, 42 cases of NATs and 19 cases of fibroadenomas (FBAs) were collected at Sun Yat‐sen University Cancer Center (SYSUCC) from 2000 to 2021 to analyse the PMR of CDO1 by MethyLight, of which, 14 BC tissues and 17 NATs were used to evaluate CpG methylation status by using MassARRAY mass spectrometry methylation sequencing analysis. The cohort II of serum samples from 113 BC patients, 31 normal people and 37 patients with benign breast disease were collected at SYSUCC from 2021 to 2022 to analyse the PMR of CDO1 by MethyLight.

The clinico‐pathological information for cohorts I and II is listed in Supporting Information Tables S1 and S2. The clinical information, including patient age, pathologic grade, tumour‐node metastasis (TNM) stage, ER status, PR status, HER2 status, molecular phenotype and the level of four serum biomarkers (CA125, CA153, CA199 and CEA) at diagnosis, was obtained from each patient's medical record. Patients were diagnosed based upon the Chinese Society of Clinical Oncology clinical practice guidelines (updated version, 2022). BC staging was defined by the American Joint Committee on Cancer systematic guidelines, and early‐stage BC was defined as T_0‐2_N_0‐1_M_0_/T3N0M0. HER2 positive status was defined as HER2 immunohistochemistry (IHC) 3+ or both HER2 IHC 2+ and HER2 FISH +. Molecular phenotypes were classified as Luminal A (grade 1/2, ER/PR+, HER2‐, Ki‐67 < 20%), Luminal B (B1: grade 3, ER/PR+, HER2‐, Ki‐67 ≥ 20%; B2: grade 3, ER/PR+, HER2+), HER2‐enriched (ER‐, PR‐, HER2+) and basal‐like (ER‐, PR‐, HER2‐).^28^ The cut‐off values for serum biomarkers CA125, CA153, CA199 and CEA at diagnosis were 35 U/mL, 25 U/mL, 35 U/mL and 5 ng/mL, respectively. These patients were followed until July 2022 to analyse the results. Raw clinical data for all samples are present in Supporting Information Table S3.

### 2.3 DNA isolation and bisulphite conversion

The detailed information is listed in Supporting Information File S1.

### 2.4 DNA methylation sequencing analysis

Fourteen BC samples and seventeen NAT samples from cohort I were used for DNA methylation sequencing analysis. The CDO1 promoter methylation sequencing primers are shown in Supporting Information Table S4, which generates a 351 bp PCR fragment (+18 to −332). Other detailed information is listed in Supporting Information File S1.

### 2.5 MethyLight assay

We used Oligo 7 software to design a TaqMan probe and two primers for each gene of bisulphite‐converted DNA. CDO1 primers and probes are designed to include CG sites for specific amplification of methylation sites, which generates a 109 bp PCR fragment (−142 to −250). ACTB is designed from a CpG dinucleotide‐free sequence to allow identical amplification at the methylation level. The input DNA was normalized using ACTB as an endogenous reference gene. The primer and probe information of the MethyLight are shown in Supporting Information Table S5. Other detailed information is listed in Supporting Information File S1.

### 2.6 Immunohistochemistry

Twenty‐one cases of CDO1 hypermethylated BC samples and twelve hypomethylated NATs samples were selected from cohort I with tissue availability for CDO1 IHC detection (Supporting Information Table S6). The antibody information is shown in Supporting Information Table S7. Further details are provided in Supporting Information File S1.

### 2.7 Cell culture and chemicals

The detailed information is listed in Supporting Information File S1.

### 2.8 Construction of CDO1‐overexpressing and CRISPR/dCas9‐Tet1CD plasmid

CDO1 overexpressing plasmid, which was purchased from Guangzhou Youming Biotechnology Ltd., was created by cloning CDO1 cDNA from healthy human leukocytes into the pLV‐CMV‐MCS‐3FLAG‐IRES‐Puro vector. Primers are listed in Supporting Information Table S8. The targeted demethylated vector (pLentiCRISPR/dCas9‐Tet1CD) was generously provided by Prof. Jianyong Shao (Sun Yat‐sen University, Guangzhou, China).^21^ Three sgRNA targeting CDO1 promoter and a control sgRNA (non‐specific) designed by using the CRISPOR online tool^29^ (sequence for sgRNAs is listed in Supporting Information Table S9) were cloned into the vector by using T4 polynucleotide kinase (NEB, Beijing, China), FastDigest BsmBI (ThermoFisher) and Quick Ligation Kit (NEB). Then, the corresponding lentiviruses were used to infect BC cells.

### 2.9 Quantitative real‐time PCR analysis (qRT‐PCR)

The relevant qRT‐PCR primers are shown in Supporting Information Table S10. Other detailed information is listed in Supporting Information File S1.

### 2.10 Western blot analysis

The information of antibodies employed in this study is listed in Supporting Information Table S7. Further details are provided in Supporting Information File S1.

### 2.11 CCK‐8 assay, colony‐forming assay and transwell assay

The detailed information is listed in Supporting Information File S1.

### 2.12 In vivo animal experiment

Female BALB/c nude mice (4–5 weeks, weighing 18−21 g) were obtained from Guangdong Yaokang Biotechnology, Ltd. (Guangzhou, China). Further details are provided in Supporting Information File S1.

### 2.13 RNA sequencing

RNA sequencing (RNA‐seq ) were performed by CapitalBio Technology (Beijing, China). Please refer to the previous research report for details about the specific experimental method.^30^, ^31^, ^32^ Further details are provided in Supporting Information File S1.

### 2.14 Cell cycle

CytoFLEX S flow cytometer (Beckman Coulter, Inc.) was used to measure the red fluorescence of 10 000 cells at 488 nm, and then CytExpert 2.3 (Beckman Coulter, Inc.) was used to analyse the results.^33^ Further details are provided in Supporting Information File S1.

### 2.15 Cell apoptosis

Cell apoptosis rate = (early apoptosis + late apoptosis)/total number of cells per well.^33^ Further details are provided in Supporting Information File S1.

### 2.16 Measurement of lipid ROS and intracellular iron levels

C11‐BODIPY (Thermo Fisher) was used to detect lipid reactive oxygen species (ROS). Total intracellular iron and ferrous iron (Fe^2+^) were measured using an iron assay kit (Sigma–Aldrich). Further details are provided in Supporting Information File S1.

### 2.17 Statistical analysis

The statistical analysis of TCGA data was performed using R (version 3.6.3). Other data were analysed using SPSS26.0 (SPSS). Any two groups were compared using the *t*‐test or the Mann–Whitney U test. One‐way ANOVA or the Kruskal–Wallis H test were used for multiple comparisons. Spearman's correlation coefficients were used to assess the correlation between CDO1 gene expression and methylation level. The relationship between CDO1 promoter methylation and clinic‐pathological parameters was analysed by χ^2^ test or Fisher's exact test. ROC analysis was used to describe the diagnostic accuracy. AUCs were reported including 95% CIs. Survival curves were constructed using the Kaplan–Meier method and compared using the log‐rank test. Cytological experiments were repeated two to three times. All data were presented as mean ± standard deviation (SD). Statistical significance was set as *p* < .05 in a two‐tailed test.

## 3 RESULTS

### 3.1 Hypermethylation of the CDO1 promoter in BC tissues predicted a poor prognosis of the patients

First, the bioinformatics analysis was performed using a publicly available database. Based on TCGA data, we compared CDO1 expression levels between 24 types of human cancer tissues and normal samples. The analysis revealed down‐regulation of CDO1 expression in 17 types of human cancers including BC (Figure 1B, Supporting Information Figure S1A). Subsequently, we analysed CDO1 methylation status in both primary BC tissues and unpaired NATs from TCGA data. Ten CpG sites located within the CDO1 promoter region (location information listed in Supporting Information Table S11) exhibited significant hypermethylation in BC tissues, compared to NATs (Figure 1C). Furthermore, analysis using the UALCAN database also confirmed hypermethylation of the CDO1 promoter region in BC (Figure 1D). Intriguingly, hypermethylation of all 10 CpG sites correlated with a decrease in CDO1 mRNA expression (Figure 1E, Supporting Information Figure S1B). Notably, the hypermethylation status of the cg16707405 site and the cg07405021 site was linked to worse OS in BC patients (Figure 1F). Moreover, CDO1 hypermethylation of cg16707405 site and cg07405021 site was associated with ER+, HER2+ and non‐basal subtype molecular phenotypes (Supporting Information Figure S1C–F).

**FIGURE 2:**
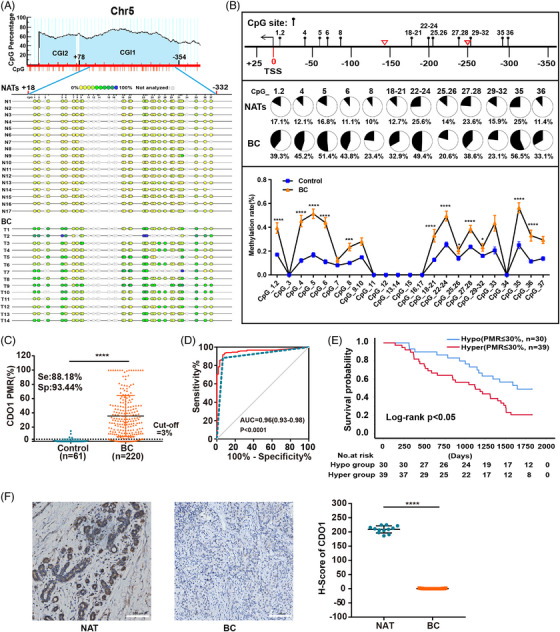
Hypermethylation of the CDO1 promoter in BC tissues and its correlation with poor prognosis. (A) Prediction of the CpG‐rich region within the CDO1 promoter using MethPrimer. CGI1 contains multiple CpG sites and is located in the region from +78 to −354 (top). MassARRAY mass spectrometry methylation sequencing was performed on the CDO1 promoter from +18 to −332 in BC tissues (n = 14) and NATs (n = 17) (bottom). This region consists of 23 CpG sites (amplicons) and 37 individual CpGs. The yellow–green–blue colour‐graded scale represents the range of DNA methylation levels, ranging from low to high (yellow = 0% and blue = 100% methylation). (B) Diagram illustrating the hypermethylated CpG sites in CGI1 based on the methylation sequencing data (top). The red triangle indicates the design site of MethyLight primers. Each lollipop represents a CpG amplicon. The pie chart shows the average PMR of each hypermethylated CpG site in NATs and BC (middle). The line chart displays the difference in CpG methylation rates between NATs and BC (bottom). A total of 19 CpG sites were accurately sequenced by MassARRAY, excluding L‐mass/H‐mass, D, OL and SN/SM amplicons, such as CpG_7, CpG_9.10, CpG_33 and CpG_37 (detailed information in Section 2). (C) Presentation of the PMR in BC tissues (n = 220) and NATs (n = 61). (D) ROC curve depicting the PMRs' ability to distinguish between BC patients and non‐cancer patients. (E) Kaplan–Meier analysis of the 5‐year PFS in BC patients grouped as hypermethylated (PMR > 30%) and hypomethylated (PMR ≤ 30%). Log‐rank test: *p* < .05. (F) Immunohistochemistry (IHC) analysis of CDO1 in BC tissues and NATs (magnification 20×, scale bar: 100 μm). Student's *t*‐test (two‐sided) was used for group comparisons, and the data are presented as mean ± SD. *p < .05, ***p < .001, **** *p* < .0001. ROC, receiver operating characteristic curve; BC, breast cancer; PMR, percent of methylated reference; Se, sensitivity; Sp, specificity; AUC, area under the curve; Hyper, hypermethylation; Hypo, hypomethylation.

To validate these findings, a MassARRAY mass spectrometry methylation sequencing analysis was performed. MethPrimer predicted the presence of two CGIs (CGI1 and CGI2) within the CDO1 promoter.^34^, ^35^ Considering that CGI1 (+78 to −354) encompasses the transcription start site and contains more CpG sites, we focused on analysing the methylation status of the region within CGI1 (+18 to −332) (Figure 2A, top). Fourteen BC tissues and seventeen unpaired NATs were subjected to MassARRAY mass spectrometry methylation sequencing to evaluate the methylation status of this region (Figure 2A, bottom). A total of 23 amplified fragments (CpG_1.2, CpG_3, CpG_4, CpG_5, CpG_6, CpG_7, CpG_8, CpG_9.10, CpG_11, CpG_12, CpG_13.14, CpG_15, CpG_16.17, CpG_18.19.20.21, CpG_22.23.24, CpG_25.26, CpG_27.28, CpG_29.30.31.32, CpG_33, CpG_34, CpG_35, CpG_36, CpG_37) were detected in this region, encompassing a total of 37 CpG sites (Figure 2A bottom, Figure 2B). EpiTYPER software accurately analysed 19 CpG sites in CGI1, of which 12 CpG sites exhibited heavy methylated in BC tissues (CpG_1.2: 39.3%, CpG_4: 45.2%, CpG_5: 51.4%, CpG_6: 43.8%, CpG_8: 23.4%, CpG_18.19.20.21: 32.9%, CpG_22.23.24: 49.4%, CpG_25.26: 20.6%, CpG_27.28: 38.6%, CpG_29.30.31.32: 23.1%, CpG_35: 56.5% and CpG_36: 33.1%, respectively), while NATs showed relatively lower methylation levels (17.1, 12.1, 16.8, 11.1, 10, 12.7, 25.6, 14, 23.6, 15.9, 25 and 11.4%, respectively) (Figure 2B). Four CpG sites (CpG_7, CpG_9.10, CpG_33 and CpG_37) were excluded as they could not be accurately recognized by EpiTYPER software. The location information of these 12 hypermethylated CpG sites in CDO1 promoter is listed in Supporting Information Table S12.

Based on the aforementioned 12 hypermethylated CpG sites identified through methylation sequencing, and considering the design requirements of PCR primers and probes, we developed a MethyLight assay. This assay amplifies a 109‐bp PCR fragment (from −142 to −250) containing 15 individual CpG sites (location information listed in Supporting Information Table S13) to measure the PMR of these 15 CpG sites in the CDO1 promoter. Then the PMR of the CDO1 promoter was determined in cohort I, which consisted of 220 BC tissues and 61 control samples containing 42 NATs and 19 FBAs using the MethyLight. The PMR in BC tissues (35.51% ± 28.74) was markedly higher than that in the NATs (.90% ± 2.68, *p* < .0001) and FBAs (.62% ± 2.35, *p* < .0001) (Figure 2C, Supporting Information Figure S2A). ROC analysis showed the high diagnostic efficacy of PMR for BC with an area under the ROC (AUC) of .96 (95% CI: .93–.98), 88.18% sensitivity, 93.44% specificity and the optimal cut‐off value of 3% (Figure 2D). Among the total of 220 BC patients in cohort I, only 69 patients had complete follow‐up data. These 69 patients were stratified into hypermethylated and hypomethylated groups based on the median PMR of 30%. The outcome analysis showed that the 5‐year PFS rate of the hypomethylated group was significantly higher than that of the hypermethylated group (48.8 vs. 20.5%, *p* < .05) (Figure 2E). In addition, high PMR of the CDO1 promoter was correlated significantly with the ER status, PR status, advanced T stage, and molecular phenotypes of non‐basal type (*p* < .05), but not with HER2 status and HER2‐enriched subtype (Table 1 and Supporting Information Figure S2B–G). IHC analysis showed that CDO1 expression was nearly absent in hypermethylated BC tissues (n = 21), whereas it increased in hypomethylated NATs (n = 12), with H‐scores of .52 ± .60 and 209 ± 12.5, respectively (*p* < .0001) (Figure 2F). Taken together, we confirmed the frequent hypermethylation of the CDO1 promoter in BC tissues, which correlated with a poor prognosis for BC patients.

**TABLE 1:** Relationship between the PMR of the CDO1 promotor and clinic‐pathologic parameters of 220 BC tissues.

		PMR, n (%)			
Characteristic	Number of cases, %	PMR ≤ 30%	PMR > 30%	χ2	*p* Value
Age (years, n%)				2.561	.109
≤50	120 (54.5)	67 (55.8)	53 (44.2)		
>50	100 (45.5)	45 (45.0)	55 (55.0)		
TNM stage				.606	.436
I–II	126 (57.3)	67 (53.2)	59 (46.8)		
III–IV	94 (42.7)	45 (47.9)	49 (52.1)		
T stage				14.556	<.05
T1–2	187 (85.0)	105 (56.1)	82 (43.9)		
T3–4	33 (15.0)	7 (21.2)	27 (81.8)		
N stage				.533	.465
N0	62 (28.2)	34 (54.8)	28 (45.2)		
N1–3	158 (71.8)	78 (49.4)	80 (50.6)		
M stage				.514	.473
M0	177 (80.5)	88 (49.7)	89 (50.3)		
M1	43 (19.5)	24 (55.8)	19 (44.2)		
Pathological grade				.023	.879
I–II	117 (53.2)	59 (50.4)	58 (49.6)		
III	103 (46.8)	53 (51.5)	50 (48.5)		
ER				9.44	<.05
Negative	57 (25.9)	39 (68.4)	18 (31.6)		
Positive	163 (74.1)	73 (44.8)	90 (55.2)		
PR				7.722	<.05
Negative	92 (41.8)	57 (62.0)	35 (38.0)		
Positive	128 (58.2)	55 (43.0)	73 (57.0)		
HER‐2 (IHC)				.138	.71
Negative	144 (65.5)	72 (50.0)	72 (50.0)		
Positive	76 (34.5)	40 (52.6)	36 (47.4)		
Molecular phenotypes				13.992	<.05
Luminal A	32 (14.5)	17 (53.1)	15 (46.9)		
Luminal B	133 (60.5)	56 (42.1)	77 (57.9)		
HER2‐enriched	28 (12.7)	18 (64.3)	10 (35.7)		
Basal‐like	27 (12.3)	21 (77.8)	6 (22.2)		

*p* values were calculated using χ2 test, or Fisher's exact test.

Abbreviations: ER, estrogen receptor; HER2, human epidermal growth factor receptor; PR, progesterone receptor; PMR, percent of methylated reference; TNM, tumour‐node‐metastasis.

Previous studies have indicated that DNA methylation inhibits gene transcription through two main mechanisms^36^: (1) DNA methylation can prevent transcription regulators from recognizing their homologous target sequences. (2) It can recruit transcription factors that specifically bind to methylated DNA, which are collectively known as methyl‐CPG binding proteins. To explore the potential transcription factors that are involved in CDO1 regulation, we predicted 13 transcription factors in the CGI1 region of CDO1 promoter by UCSC Genome Browser on Human (GRCh38/hg38) (Supporting Information Figure S3A), and furthermore, 7 transcription factors were identified with specific binding sites within the CGI1 region of the CDO1 promoter by the JASPAR online tool (Supporting Information Figure S3B, Table S14). Of these 7 transcription factors, the binding sites of ZNF263, SP2, KLF12, PATZ1, E2F6 and ZNF281 contained hypermethylated CpG sites in the CDO1 promoter in BC (Supporting Information Table S12). Notably, TCGA data analysis revealed correlations between the expression of ZNF263, SP2, and ZNF281 and CDO1 expression in hypomethylated BC samples, which were absent in hypermethylated samples (Supporting Information Figure S3C). Given that these transcription factors belong to the C2H2 zinc finger protein family, known for binding methylated DNA to repress gene transcription,^37^, ^38^, ^39^ we hypothesize that ZNF263, SP2 and ZNF281 may collaborate with hypermethylated CpG sites to inhibit CDO1 expression (Supporting Information Figure S3D), and further research is required to elucidate the specific underlying mechanisms.

### 3.2 CDO1's role as a tumour suppressor in BC

**FIGURE 3:**
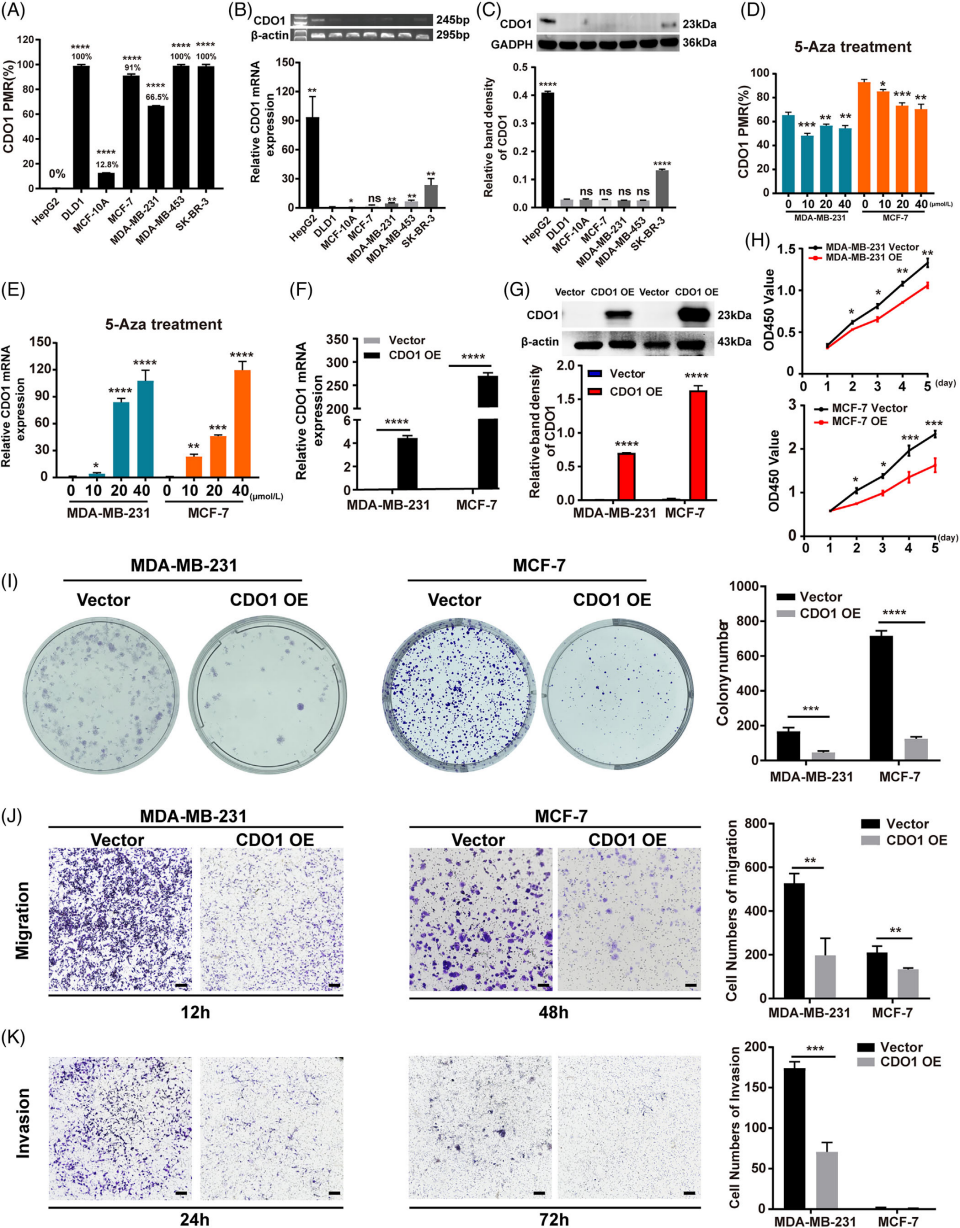
CDO1 downregulation due to promoter hypermethylation and its role as a tumour suppressor gene in BC. (A) PMR of CDO1 in various cell lines: MCF‐7, MDA‐MB‐231, MDA‐MB‐453, SK‐BR‐3, MCF‐10A, HepG2 and DLD1. (B) CDO1 mRNA levels in the mentioned cell lines, assessed using Agarose gel electrophoresis (top) and RT‐PCR (bottom). (C) Protein levels of CDO1 in the mentioned cell lines. Raw immunoblots are included in Supporting Information Figure S4. (D) PMR of CDO1 promoter in MDA‐MB‐231 and MCF‐7 cells treated with 5‐Aza (0, 10, 20 and 40 μM) for 4 days. (E) CDO1 mRNA levels in two BC cell lines treated with 5‐Aza for 4 days. (F) CDO1 mRNA levels in two BC cell lines with or without CDO1 overexpression. (G) Protein levels of CDO1 in two BC cell lines with or without CDO1 overexpression. Raw immunoblots are included in Supporting Information Figure S6. (H) Percentage of viable BC cells with or without CDO1 overexpression, measured as absorbance (OD450 value). (I) Number of colonies formed by MDA‐MB‐231 and MCF‐7 cells with or without CDO1 overexpression. (J) Number of migratory MDA‐MB‐231 and MCF‐7 cells with or without CDO1 overexpression. Left panel: representative image of the Transwell assay (magnification 10×, scale bar: 500 μm); right panel: quantitative analysis. (K) Number of invasive BC cells with or without CDO1 overexpression. Left panel: representative image of the Transwell assay (magnification 10×, scale bar: 500 μm); right panel: quantitative analysis. Data presented as mean ± SD of three independent experiments. *p < .05, **p < .01, ***p < .001, ****p < .0001. Statistical analysis: two‐sided Student's *t*‐test. OE represents overexpression of CDO1. Vector represents negative controls.

Based on the aforementioned findings, we hypothesized that CDO1 promoter hypermethylation plays a crucial role in BC development by silencing CDO1 expression. To investigate this, we examined the methylation level and expression level of CDO1 in four BC cell lines (MDA‐MB‐231, MCF‐7, MDA‐MB‐453 and SK‐BR‐3) and a normal breast cell line (MCF‐10A). As a positive control, DLD1 (colorectal cancer cell line) and HepG2 (hepatocellular cancer cell line) cells, known to have methylation rates of 93% and 4.7%, respectively, in the CpG sites of the CDO1 promoter region, were included.^40^ Our results showed that the CDO1 promoter was hypermethylated in four BC cell lines (*p* < .0001, Figure 3A), along with significantly decreased mRNA and protein expression of CDO1 (Figure 3B, C, Supporting Information Figure S4). Notably, although the CDO1 PMR was relatively low in MCF‐10A cells, the CDO1 mRNA and protein expression levels were almost absent. Since the MethyLight assay measured PMR of the average methylation status of these 15 CpG sites in CDO1 promoter (from −142 to −250) and the CDO1 promoter region has two CGIs (Figure 2A). Hence, we highly speculated that CDO1 may be hypermethylated in the other regions of the CDO1 promoter to repress gene expression in MCF‐10A. Further analysis using MassARRAY mass spectrometry methylation sequencing confirmed that there were hypermethylated modification sites in the +47 to +382 bp region of the CDO1 promoter in MCF‐10A cells (Supporting Information Figure S5). However, it remains to be further verified whether the low expression of CDO1 in MCF‐10A is caused by the hypermethylation of these specific CpG sites.

**FIGURE 4:**
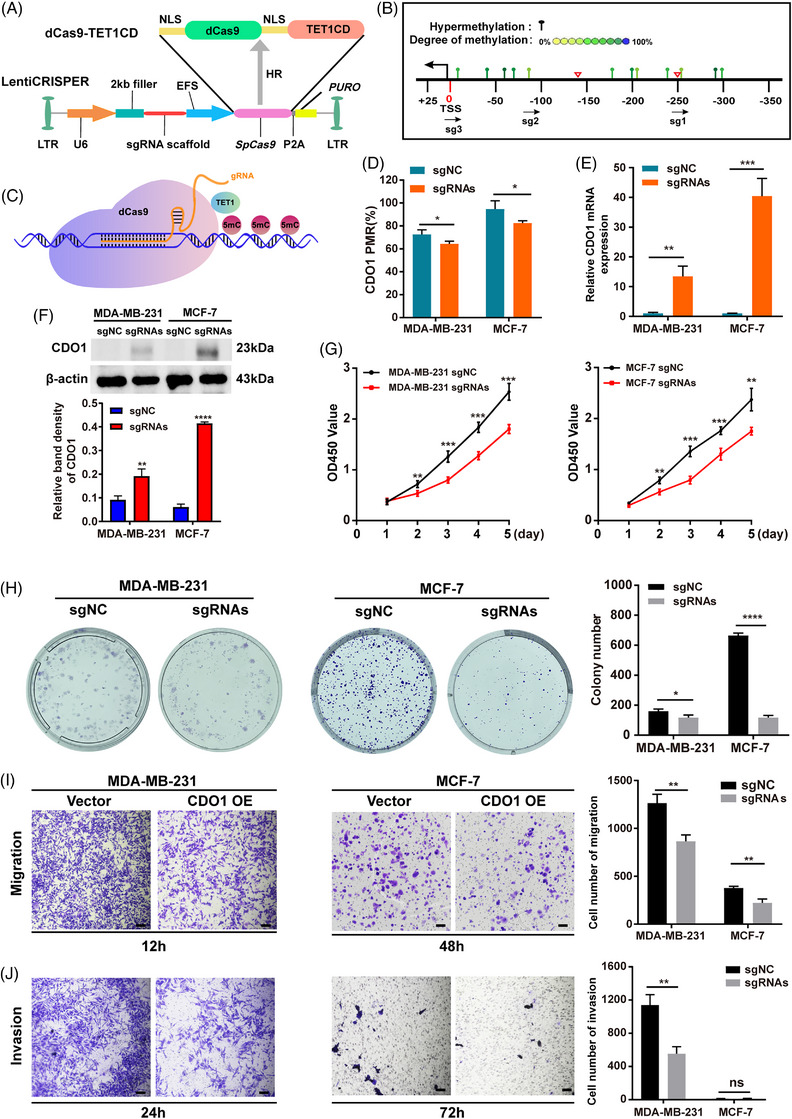
Targeted demethylation at the CDO1 promoter induced CDO1 expression and inhibited the proliferation and migration of BC cells in vitro. (A) Schematic diagram illustrating the CRISPR/dCas9‐TET1/CD demethylation plasmid. (B) Three sgRNAs were designed to target the hypermethylation region of the CDO1 promoter (indicated by the arrow). The 3ʹ end of the sgRNAs was represented by the arrowhead. The DNA methylation levels are plotted in a yellow–green–blue colour‐graded scale (yellow = 0% and blue = 100% methylation), with the red triangle denoting the MethyLight primer design site. (C) The CRISPR‐dCas9‐Tet1CD fusion protein was recruited to the CDO1 promoter region and facilitated the removal of hypermethylation sites under the guidance of sgRNA. (D) PMR of the CDO1 promoter in BC cells expressing sgRNAs and sgNC (negative control sgRNA). (E) CDO1 mRNA levels in BC cells expressing sgRNAs and sgNC. (F) CDO1 protein levels in BC cells expressing sgRNAs and sgNC. All raw immunoblots are included in Supporting Information Figure S7. (G) Viability of BC cells expressing sgRNAs and sgNC, expressed as absorbance (OD450 value). (H) The number of colonies formed by BC cells expressing sgRNAs and sgNC. (I) The number of migratory BC cells expressing sgRNAs and sgNC. The left panel shows representative images of the Transwell assay (magnification 10×, scale bar: 500 μm), while the right panel presents the quantitative analysis. (J) The number of invasive BC cells expressing sgRNAs and sgNC. The left panel shows representative images of the Transwell assay (magnification 10×, scale bar: 500 μm), and the right panel provides the quantitative analysis. Data presented are the mean ± SD of three independent experiments. Statistical analysis was performed using Student's *t*‐test (two‐sided). ns: *p* ≥ .05, * *p* < .05, **p < .01, ***p < .001, ****p < .0001. sgRNAs, The pool of sgRNA1, sgRNA2 and sgRNA3; 5mC, 5‐methylcytosine; sgNC, sgRNA for negative control; U6 promoter; EFS promoter; NLS, nuclear localization signal; Puro, puromycin; SpCas9, *S. pyogenes* Cas9; LTR, long terminal repeat.

Since MDA‐MB‐453 cells grown in semi‐suspension are unsuitable for adherence‐related functional experiments, they were excluded from further analyses. In contrast, MCF‐7 (estrogen receptor‐positive cells) and MDA‐MB‐231 cells (triple‐negative cells) have good adherence and demonstrate a high rate of tumour formation in vivo.^41^, ^42^, ^43^ Additionally, SK‐BR‐3 cells showed a low level of CDO1 expression (Figure 3B, C). Consequently, MCF‐7 and MDA‐MB‐231 cell lines were selected for subsequent experiments. Treatment of these cell lines with 5‐Aza, a potent DNA methyltransferase inhibitor, resulted in a reduction in the PMR of the CDO1 promoter and a concurrent upregulation of CDO1 mRNA (Figure 3D, E). To further validate CDO1's tumour suppressive function, we employed CDO1 overexpression strategy in MDA‐MB‐231 and MCF‐7 cells, which demonstrated that CDO1 overexpression significantly inhibited cell proliferative, cell migrative and invasive capacities in vitro compared to the controls (Figure 3F–K, Figure S6). For further validation, a targeted demethylation technology based on CRISPR/Cas9 system^21^ (Figure 4A) was utilized in this study. Three specific sgRNAs designed to target the CDO1 promoter, along with a non‐specific control sgRNA (sgNC), were used to construct a pLentiCRISPR‐dCas9‐Tet1CD‐sgRNAs‐based specific demethylation system (Figure 4B, Supporting Information Table S9). After the BC cells were infected by lentiviral, dCas9‐Tet1CD fusion protein was recruited by sgRNA to a specific hypermethylated region of CDO1 promoter, resulting in Tet1CD‐induced DNA demethylation (Figure 4C). Compared with the control cells, significant demethylation of the CDO1 promoter, accompanied by upregulated CDO1 mRNA and protein levels, was observed in the CRISPR‐treated BC cells (Figure 4D–F, Supporting Information Figure S7). Moreover, demethylation of CDO1 promoter restrained the proliferation, migration and invasion of BC cells in vitro (Figure 4G–J).

**FIGURE 5:**
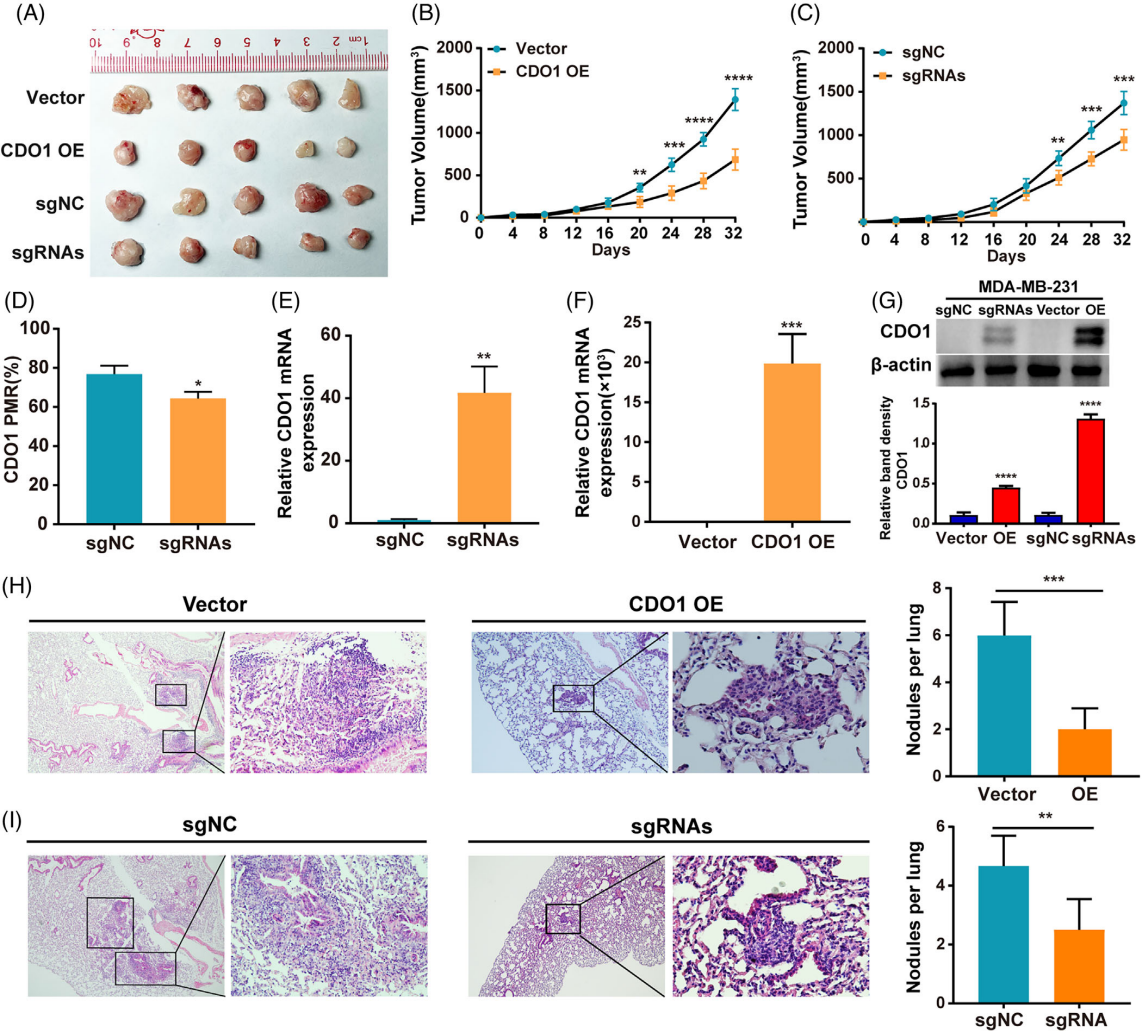
Targeted demethylation strategy inhibited tumour growth of BC cells in vivo. (A) Representative image of the tumour derived from MDA‐MB‐231 cells with both targeted demethylation of the CDO1 promoter and CDO1 overexpression. (B and C) Tumour growth curves of the indicated groups. (D) PMR of CDO1 following targeted demethylation. (E–G) CDO1 mRNA and protein levels in the demethylation and overexpression groups. All raw immunoblots are included in Supporting Information Figure S8. (H & I) Representative images of HE‐stained lung metastatic nodules (magnification: left, 4×; right, 20×) and the number of metastatic nodules. Upper: Overexpression group (H). Lower: Demethylation group (I). Data presented are the mean ± SD. Statistical analysis was performed using the Student's *t*‐test (two‐sided). *p < .05, **p < .01, ***p < .001, ****p < .0001.

To assess CDO1's function in vivo, the appropriately constructed MDA‐MB‐231 cells were injected into the right scapular region of nude mice, establishing a subcutaneous xenograft model. As expected, both the CDO1 overexpression group and the CDO1 promoter‐targeted demethylation group exhibited significant growth retardation of tumours compared to the control mice (Figure 5A). The average tumour volume of xenografts tumours in CDO1‐overexpressing group and vector control group was 685.4 ± 123.3 and 1394.0 ± 128.8 mm^3^, respectively (Figure 5B, *p* < .0001). The average tumour volume of xenografts tumours in CDO1 promoter‐targeted demethylation group and control group was 947.1 ± 118.8 and 1371.0 ± 134.2 mm^3^, respectively (Figure 5C, *p* < .001). The MethyLight assay confirmed that a sustained CDO1 demethylation status was induced by dCas9‐Tet1CD in vivo (*p* = .016) (Figure 5D). In addition, both CDO1 mRNA level and protein level were upregulated in the demethylated group and CDO1‐overexpressing group, compared to their respective control groups (Figure 5E–G, Supporting Information Figure S8). To establish a tumour metastasis model, BALB/c nude mice were injected with demethylated and CDO1‐overexpressing MDA‐MB‐231 cells via the tail vein. The number of metastatic lung nodules was significantly reduced in the demethylation group (*p* < .01) and overexpression group (*p* < .001) compared to the control groups (Figure 5H, I). Targeted demethylation of the CDO1 promoter in vivo restores CDO1 expression and inhibits BC cell progression. Collectively, CDO1 functions as a tumour suppressor in BC.

### 3.3 CDO1 arrested the cell cycle and induced cell death in BC

**FIGURE 6:**
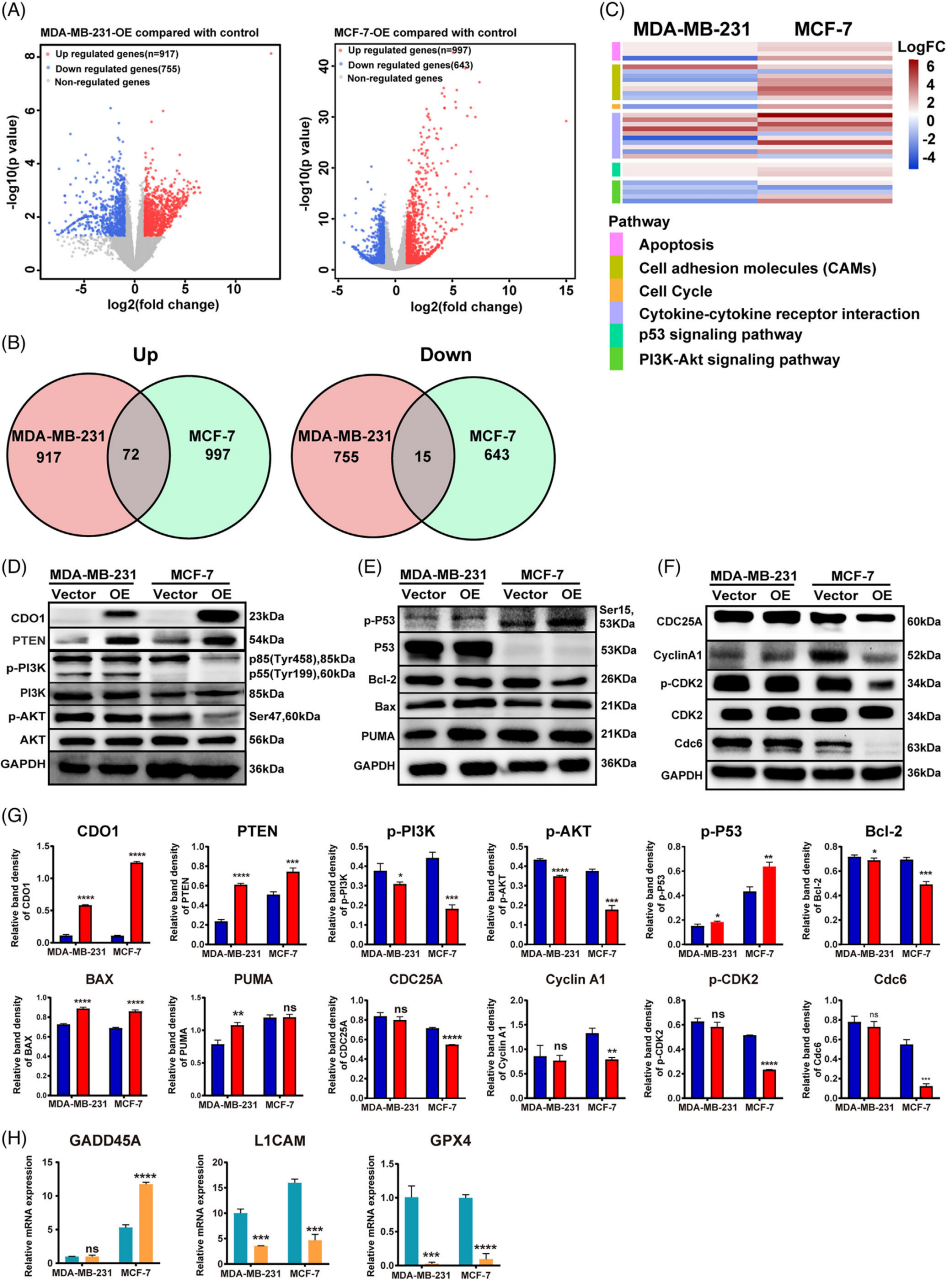
Differentially expressed genes and signalling pathways influenced by CDO1 in two BC cell lines. (A) Volcano plot showing the differentially expressed genes in BC cells with or without CDO1 overexpression. (B) Venn diagram illustrating the overlapping upregulated and downregulated DEGs in two BC cell lines stably expressing CDO1. (C) Enrichment analysis of KEGG pathways for the differentially abundant genes induced by CDO1 in two BC cell lines stably expressing CDO1. Representative protein gel bands of the PI3K‐AKT signalling pathway (D), p53 pathway (E), and cell cycle pathway (F) in two BC cell lines stably expressing CDO1. All raw immunoblots are included in Supporting Information Figure S9. (G) Relative intensity of CDO1, PTEN, p‐PI3K, PI3K, p‐AKT, AKT, p‐P53, P53, Bcl‐2, Bax, PUMA, CDC25A, Cyclin A1, p‐CDK2, CDK2 and Cdc6 in two BC cell lines stably expressing CDO1. (H) mRNA levels of GADD45A, L1CAM and GPX4 in two BC cell lines stably expressing CDO1. Data are presented as mean ± SD of three independent experiments. ns *p* > .05, *p < .05, **p < .01, ***p < .001, ****p < .0001. Statistical analysis was performed using the Student's *t*‐test (two‐sided).

The RNA‐seq analysis was used to evaluate the differentially expressed genes (DEGs) in two CDO1 overexpressing BC cells (MDA‐MB‐231 and MCF‐7). We identified that 917 genes were upregulated and 755 genes were downregulated in MDA‐MB‐231 cells stably expressing CDO1 (Figure 6A left), while 997 genes were upregulated and 643 genes were downregulated in MCF‐7 cells (Figure 6A, right). The number of common DEGs in the two cell lines was 87, including 72 upregulated genes and 15 downregulated genes (Figure 6B). Then, we conducted KEGG pathway enrichment analysis on these common DEGs and revealed their involvement in the p53 pathway and apoptosis in BC cell lines (Figure 6C). Western blot analysis confirmed that CDO1 overexpression led to significant inhibition of the PI3K/AKT pathway and activation of the p53 pathway in two CDO1‐ overexpressing BC cell lines (Figure 6D, E, G, Supporting Information Figure S9). Moreover, the expression of PTEN and Bax was significantly upregulated in CDO1‐overexpressing BC cells, while BCL‐2 was obviously downregulated (Figure 6D, E, G and Supporting Information Figure S9). These results suggested that CDO1 may inhibit cell proliferation and promote cell apoptosis of BC cells by inhibiting the PI3K/AKT pathway and activating the p53 pathway. Notably, CDO1 overexpression also resulted in decreased expression of cell cycle‐related proteins such as CDC25A, cyclinA‐CDK2 complex and Cdc6 in MCF‐7 cells (Figure 6F, G and Supporting Information Figure S9). We also found that GADD45A was upregulated in MCF‐7 cells with stable expression of CDO1 (Figure 6H). Therefore, CDO1 may block the progress of cell cycle in MCF‐7 cells. Simultaneously, CDO1 could significantly downregulate the adhesion molecule L1CAM in two BC cells (Figure 6H). Furthermore, CDO1 downregulated the ferroptosis marker GPX4 in both BC cells (Figure 6H), suggesting a potential role for CDO1 in inducing ferroptosis.

**FIGURE 7:**
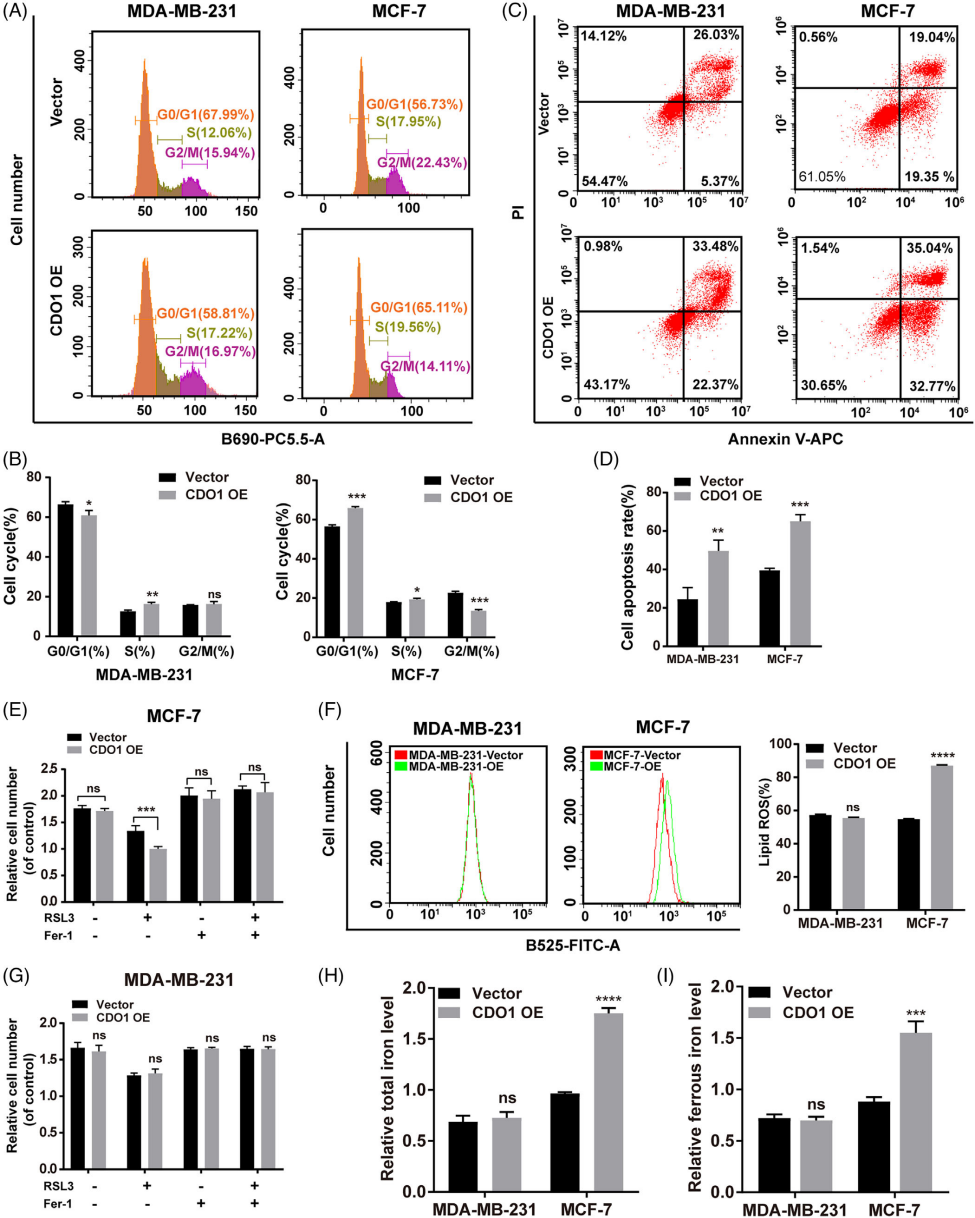
Effects of CDO1 overexpression on cell cycle progression, apoptosis and ferroptosis in BC cells. (A) Representative flow cytometry histograms illustrating the percentage of cells in different cell cycle phases in MDA‐MB‐231 and MCF‐7 cells. (B) Quantification of the percentage of cells in the three different phases of the cell cycle in MDA‐MB‐231 and MCF‐7 cells. (C) Representative flow cytometry plots demonstrating the cell apoptosis rate in MDA‐MB‐231 and MCF‐7 cells. The upper right corner of the cross gate represents late apoptotic cells, and the lower right corner represents early apoptotic cells. (D) Quantification of the cell apoptosis rate in MDA‐MB‐231 and MCF‐7 cells. (E and G) CCK‐8 assay showing the response of MCF‐7 and MDA‐MB‐231 cell lines to RSL3 (3 μM) ± ferrostatin (2 μM) for 24 h. (F) Measurement of lipid ROS levels in MDA‐MB‐231 and MCF‐7 cells. (H) Quantification of total iron levels in MDA‐MB‐231 and MCF‐7 cells. (I) Quantification of ferrous iron levels in MDA‐MB‐231 and MCF‐7 cells. Data are presented as mean ± SD of three independent experiments. ns: *p* > .05, *p < .05, **p < .01, ***p < .001, ****p < .0001. Statistical analysis was performed using the Student's *t*‐test (two‐sided).

To further investigate the possible underlying mechanism of CDO1 as a tumour suppressor in BC, cell cycle assay and cell death assay were performed in two CDO1‐overexpressing BC cells (MDA‐MB‐231 and MCF‐7). The results of the cell cycle assay revealed a notable decrease in the percentage of cells in the G0/G1 phase and a significant increase in the S phase in MDA‐MB‐231 cells that overexpressed CDO1, compared to the control group (Figure 7A, B). CDO1 overexpression may arrest the MDA‐MB‐231 cell cycle at S phase, resulting in cell growth inhibition. However, several cell cycle‐related genes that we detected in CDO1‐expressing MDA‐MB‐231 cells were not obviously changed (Figure 6F, G), and we speculated that CDO1 overexpression may arrest MDA‐MB‐231 cell cycle progression by affecting other cycle‐related proteins that we have not detected. The percentage of cells in G0/G1 phase and S phase was significantly increased, whereas that in G2/M phase was significantly decreased, in CDO1‐overexpressing MCF‐7 cells compared with that in control cells (Figure 7A, B). This indicates that CDO1 overexpression arrested the cell cycle of MCF‐7 cells at the G0/G1 phase and the S phase, inhibiting cell growth.

The apoptosis rate of BC cells overexpressing CDO1 was significantly higher than that of vector control cells (Figure 7C, D). Therefore, CDO1 overexpression promoted apoptosis of BC cells. In addition, the regulation of GPX4 by CDO1 prompted us to explore the involvement of CDO1 in ferroptosis. To do this, we evaluated RSL3‐induced ferroptosis in the presence or absence of ferrostatin, an inhibitor of ferroptosis. Here, we showed that CDO1 overexpression increased RSL3‐induced growth inhibition in MCF‐7 cells (Figure 7E). The levels of intracellular iron and lipid ROS were used as indicators of ferroptosis.^44^ In this study, we found CDO1 increased the levels of both lipid ROS and iron in MCF‐7 cells, however, no such increase was observed in MDA‐MB‐231 cells (Figure 7F–I). Collectively, our data showed that CDO1 overexpression induced ferroptosis of MCF‐7 cells.

### 3.4 Diagnostic value of serum CDO1 methylation status in early‐stage BC

**FIGURE 8:**
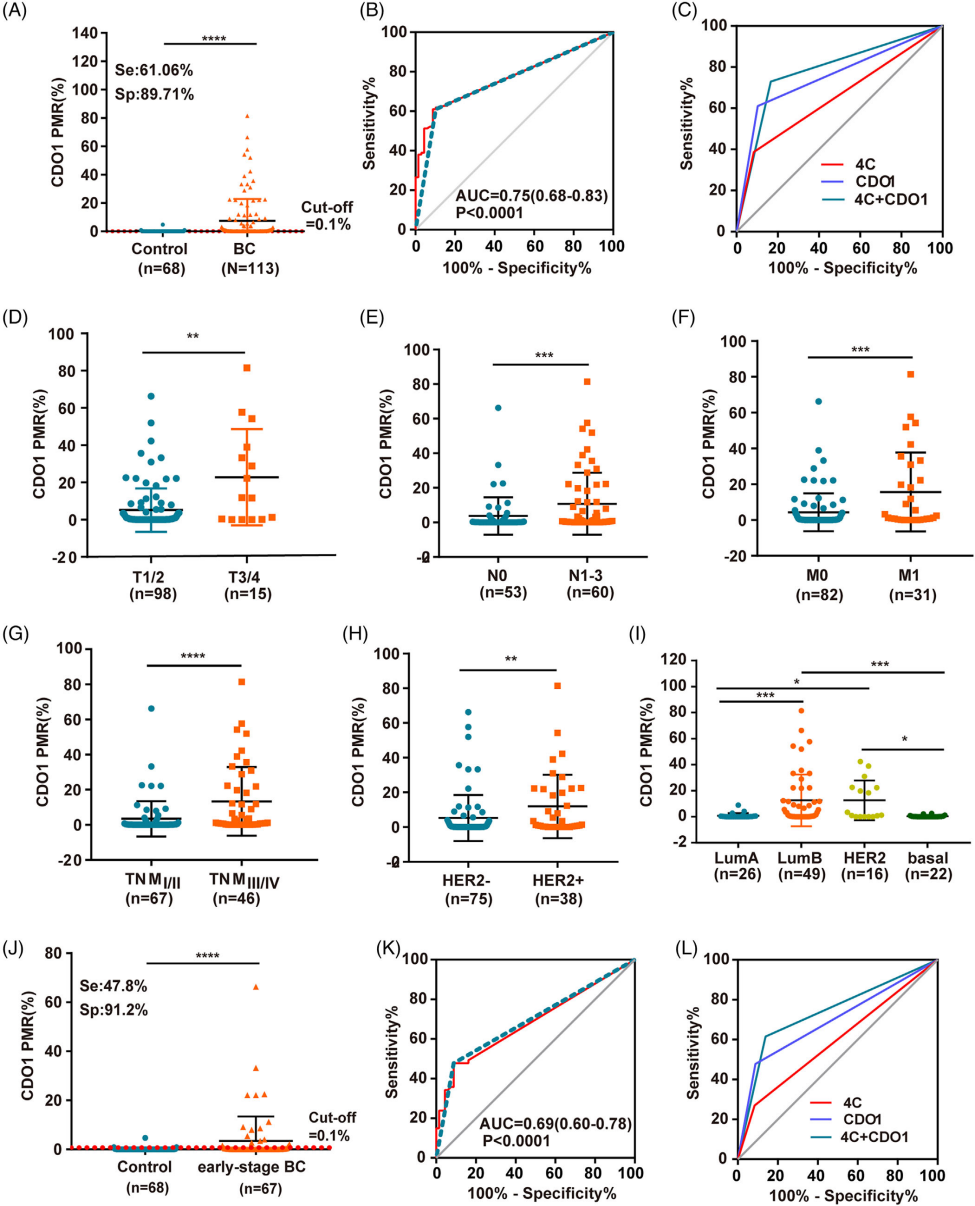
Diagnostic values of serum CDO1 methylation biomarkers and traditional tumour antigen biomarkers 4C for early‐stage BC patients. (A) PMR of serum CDO1 in control patients and BC patients. (B) ROC curve of PMRs for distinguishing between BC patients and non‐cancer patients (*p* < .0001). (C) ROC curve of CDO1, 4C and CDO1 + 4C for distinguishing between BC patients and non‐cancer patients. (D–H) PMR of serum CDO1 for T1/2 vs. T3/4, N0 vs. N1‐3, M0 vs. M1, TNMI/II vs. TNMIII/IV and HER2‐ vs. HER2+ BC patients. (I) PMR of serum CDO1 in Luminal A, Luminal B, HER2‐enriched, and basal‐like BC patients. (J) PMR of serum CDO1 in early‐stage BC (n = 67) and non‐cancer patients (n = 68). (K) ROC curve of PMRs for distinguishing between early‐stage BC patients and non‐cancer patients (*p* < .0001). (L) ROC curve of CDO1, 4C and CDO1 + 4C for distinguishing between early‐stage BC patients and non‐cancer patients. The traditional serum biomarker 4C includes CEA, CA199, CA125 and CA153. Student's *t*‐test (two‐sided) was performed to compare two groups, and the data are presented as mean ± SD. **p < .01, ***p < .001, ****p < .0001.

Given the potential of serum methylation biomarkers as non‐invasive tools for cancer screening and prognosis prediction, we evaluated the early diagnostic value of serum CDO1 for BC. First, to confirm the presence of hypermethylation in the CDO1 promoter region in serum cfDNA of BC patients, we performed the MethyLight assay to measure the PMR of CDO1 in cohort II, which included serum samples from 113 BC patients (stages I–IV) and 68 control patients (31 normal individuals and 37 patients with breast benign diseases). The PMR of CDO1 promoter in BC patients (7.46% ± 15.38) was significantly higher than that in the normal people (.19% ± .83, *p* < .0001) and patients with breast benign diseases (.04% ± .15, *p* < .0001) (Figure 8A, Supporting Information Figure S2H). Additionally, we examined the correlation between CDO1 methylation in tissue and blood samples by analysing 24 BC tissue samples from cohort II. We found that CDO1 methylation in tissue and the blood stream were highly correlated (*R* = .48, *p* = .0002, Supporting Information Figure S10), suggesting serum CDO1 methylation status may reflect the CDO1 methylation status in BC tissues.

The diagnostic efficacy of PMR for BC was found to be better through ROC analysis, with an AUC of .75 (95% CI: .68–.83), sensitivity of 61.06%, a specificity of 89.71% and an optimal cut‐off value of 0.1% (Figure 8B). In comparison, traditional serum biomarkers 4C (CEA, CA199, CA125 and CA153) exhibited a sensitivity of 38.68%, a specificity of 91.67% and an AUC of 0.65 (95% CI: .56–.75). The performance of the CDO1 methylation biomarker in differentiating the BC cases from controls was better than that of traditional tumour antigen markers. More importantly, the combination of CDO1 and 4C achieved 72.97% sensitivity and 83.33% specificity with an AUC of .78 (95% CI: 0.70–0.87) (Figure 8C). In addition, serum CDO1 hypermethylation was correlated significantly with the advanced T stage, the advanced N stage, metastasis, the TNM stage, HER2 status and molecular phenotypes in the cohort II of 113 BC patients (*p* < .05) (Supporting Information Table [Link], [Link], Figure 8D–I).

To evaluate the value of early diagnosis based on serum CDO1 methylation status, we compared the PMR of serum CDO1 between early‐stage BC patients (stages I‐II, n = 67) and control patients (n = 68). The PMR in early‐stage BC patients (3.40% ± 10.02) was significantly higher than that in the control patients (.11% ± .58, *p* < .0001) (Figure 8J). As a diagnostic biomarker, the sensitivity, specificity and AUC were 47.76, 91.18 and 0.69 (95% CI: 0.60–0.78), respectively, with the optimal cut‐off value of .1% in early‐stage BC (Figure 8K). In comparison, traditional serum biomarkers 4C exhibited a sensitivity of 26.98%, a specificity of 91.67% and an AUC of .60 (95% CI: .48–.71) for early‐stage BC. Notably, in the early BC group, the diagnostic value of serum CDO1 methylation surpassed that of 4C. Furthermore, when combining 4C and the serum CDO1 biomarker, the diagnostic efficiency improved, with a sensitivity of 61.54%, a specificity of 86.11% and an AUC of .74 (95% CI: .64–.84) (Figure 8L).

## 4 DISCUSSION

Epigenetic changes have been identified in all types of human cancers and are known to work in conjunction with genetic changes to promote the progression of cancer.^45^ Tumourigenesis involves extensive DNA methylation changes.^46^ Typically, the DNA hypermethylation of TSGs silences the gene expression, while the hypomethylation of oncogene DNA activates gene expression.^14^ In this study, there are several methods used to detect methylation status. First, the methylation data from TCGA was generated by the Illumina Human Methylation 450 BeadChip array, where each probe mostly corresponds to one CpG site, and the data represent the methylation level of each individual CpG site.^47^ The MassARRAY EPiTYPER technology combines base‐specific enzyme cleavage and MALDI‐TOF detection principles, enabling quantitative analysis and detection of multiple CpG sites with high accuracy, high throughput and high sensitivity.^48^, ^49^ However, this technology has limitations, such as difficult operation, a cumbersome process and high cost, which restrict its widespread application. MethyLight assay is a TaqMan probe‐based fluorescent quantitative polymerase chain reaction that relies on the hybridization and cleavage of probes against target CpG sequences.^50^ This assay reflects the overall PMR of all CpG sites within the amplified PCR fragment. Compared with other high‐throughput sequencing technologies, the detection cost is relatively low, and it is easy to do batch detection on a large scale of samples with high repeatability.^51^, ^52^

DNA methylation has been identified as a potential target for cancer therapy.^20^, ^21^ Traditional demethylation agents, such as 5‐Aza, act as DNA methyltransferase inhibitors, leading to DNA demethylation by covalently capturing DNA methyltransferase.^53^, ^54^ However, 5‐Aza lacks specificity, which can result in the restoration of abnormal methylation patterns in normal cells and disrupt normal gene regulation mechanisms. Targeted demethylation technology based on the CRISPR/Cas9 system can demethylate specifically the target gene without affecting other genes.^19^, ^22^ So far, there are only few reports published on epigenetic editing of BC‐related genes in BC.^55^, ^56^ P Castelo‐Branco et al. developed a CRISPR‐dCas9‐TET1‐based and peptide repeat‐based system for demethylation of the hTERT gene promoter in BC.^55^ However, the plasmid only contains fluorescent protein tags, and the constructed demethylated cells need to be obtained by flow cytometry multiple times. Based on the Tet1‐dCas9 targeted demethylation system, CA Sweeney et al. successfully demethylated the LRIG1 gene and restored its endogenous expression in triple‐negative BC cells.^56^ However, its demethylation effect is transient, and the demethylation effect in vivo could not be assessed. The demethylation system used here was adapted from Zhang et al.’s LentiCRISPR plasmid, which can constitutively express sgRNA along with Cas9 protein to achieve stable gene knockout.^21^ In addition, the puromycin gene in this plasmid allows rapid selection of stably infected cells.^57^ Consistent with the experimental results of 5‐Aza treatment, our targeted demethylation system reduced CDO1 promoter PMR by 8% and 12% in vitro and in vivo, respectively. Furthermore, the targeted demethylation strategy resulted in a significant upregulation of CDO1 mRNA levels by 13‐fold in vitro and 41‐fold in vivo, indicating its long‐lasting effects. In this study, we observed that CDO1 PMR decreased slightly, but the CDO1 mRNA level and protein level recovered significantly. This phenomenon can be attributed to the following reasons: (i) The catalysis of 5mC to 5hmC by Tet1 is the rate‐limiting step in the demethylation process. Consequently, there is a certain accumulation of 5hmC during demethylation. However, the bisulphite conversion assay used in this study is unable to distinguish between 5mC and 5hmC.^58^, ^59^ Consequently, MethyLight detects both as methylated sites, leading to higher levels of CDO1 PMR detection after demethylation. (ii) Previous research has indicated that not all CpG sites in the gene promoter region are demethylated with the same efficiency by the targeted demethylation system. However, the demethylation of CpG sites that act as regulatory elements can cause significant changes in gene expression.^55^ The lack of a clear linear correlation between CDO1 methylation level and expression level in our study may be due to the fact that MethyLight primarily detects the overall methylation level of the amplified fragment.

The study explored the underlying mechanism of the anti‐tumour function of CDO1, and found that CDO1 activated the p53 signalling pathway and inhibited the PI3K/AKT signalling pathway in MDA‐MB‐231 and MCF‐7 cells. The PI3K/AKT signalling pathway is widely recognized for its crucial role in cell proliferation, apoptosis, glucose metabolism and various other physiological processes.^60^ As downstream genes of p53 pathway, BAX and PUMA play an important role in promoting apoptosis, while BCL‐2 plays a role in anti‐apoptosis. Additionally, cell cycle‐related proteins CDC25A, cyclin A1, p‐CDK2 and Cdc6 were downregulated in MCF‐7 cells stably expressing CDO1. The degradation of CDC25A can inhibit the cyclin A1‐CDK2 complex and block the cell cycle in S phase.^61^ CDC25A dephosphorylates several CDKs, regulating not only the early G1/S transition but also the late G2/M.^62^, ^63^ Cdc6 is a necessary protein to initiate DNA replication, which acts as a regulator in the early stage of DNA replication.^64^ We also found that the expression of GADD45A, which plays a crucial part in the cell cycle,^65^, ^66^ was upregulated in CDO1‐overexpressed MCF‐7 cells. CDO1 overexpression was shown to arrest MDA‐MB‐231 cells in the S phase and MCF‐7 cells in the G0/G1 and S phases, while also increasing cell apoptosis rates in both cell lines.

Interestingly, CDO1 was found to have the ability to decrease the expression of L1CAM and GPX4 in BC cells. L1CAM and its soluble forms can promote cell adhesion and migration in BC cells and are associated with a poor prognosis of TNBCs.^67^ GPX4, which uses glutathione as the substrate, can directly eliminate the hydroperoxide in the lipid bilayer and prevent the accumulation of harmful lipid ROS, which play a crucial role in the process of ferroptosis.^68^ Cysteine is a critical component in the synthesis of glutathione and serves as the limiting substrate for this process. CDO1 can catalyse the oxidation reaction of cysteine, resulting in the blockage of glutathione production. In gastric cancer, inhibiting CDO1 expression led to the restoration of glutathione levels, increased expression of GPX4, prevented ROS production, and reduced the production of malondialdehyde, which is a final product of lipid peroxidation.^69^ The restoration of CDO1 function in BC cells resulted in an increase in ROS levels, which in turn reduced cell viability and growth while increasing sensitivity to anthracycline treatment.^8^ Erastin, a ferroptosis inducer, suppressed expression of GPX4 in TNBC and upregulated expression of CDO1.^70^ In this study, CDO1 overexpression significantly inhibited RSL3‐induced activity and increased lipid ROS levels and iron levels in MCF‐7 cells, but had no significant effect on ferroptosis‐related processes and markers in MDA‐MB‐231 cells. We speculated that the ferroptosis process in MDA‐MB‐231 may be also modulated by other unknown factors, besides GPX4, which need further study to clarify. Overall, the tumour suppressor role of CDO1 in BC is complex due to the high heterogeneity of the disease, and further research is needed to investigate its mechanism in different pathological types of BC cell lines.

Currently, the number of discovered hypermethylated TSGs in BC is limited, and most of them have low diagnostic efficiency or lack of strong evidence to prove the value of clinical application, particularly in serum samples.^71^, ^72^ However, in this study, the PMR of the CDO1 promoter region in serum showed high diagnostic efficiency for BC stages I–IV with a sensitivity of 61.2%, a specificity of 89.7% and an AUC of .75 (95% CI: .68–.83) in cohort II of 113 BC patients, and it was significantly correlated with advanced T stage, advanced N stage, metastasis, TNM stage, HER2 status and molecular phenotypes (*p* < .05). Notably, we found the PMR of serum CDO1 in early‐stage BC patients (3.40% ± 10.02) was markedly higher than that in the control patients (0.11% ± 0.58, *p* < .0001). The PMR of serum CDO1 showed high early diagnostic efficiency for BC. Surprisingly, the combination of serum CDO1 PMR and traditional serum biomarkers 4C had even higher diagnostic efficiency for early BC, with a sensitivity and specificity of 61.54 and 86.11%, respectively, and an AUC of .74 (95% CI: .64−.84). As far as we know, this is the first report on the clinical application of the PMR of serum CDO1 in BC and early‐stage BC. These results suggest that serum CDO1 methylation could be a valuable biomarker for the early diagnosis and management of BC. However, due to the limitation of the cohort used in this study, such as the relatively small sample size, retrospective study, single centre and no follow‐up data, the diagnostic value of serum CDO1 methylation in BC needs further research and verification.

In summary, our work has significantly expanded our understanding of the functional role and mechanisms of CDO1 as a TSG in BC, as well as evaluating the translational potential of serum CDO1 levels for early BC diagnosis. Additionally, we have explored the application of targeted epigenetic editing in BC therapy.

## 5 CONCLUSIONS

In conclusion, CDO1 is hypermethylated and functions as a TSG in BC. Epigenetic editing of abnormal CDO1 methylation could have a crucial role in the clinical treatment of BC. Additionally, serum CDO1 methylation shows promise as a diagnostic biomarker for BC, especially in early‐stage patients.

## CONFLICT OF INTERESTS STATEMENT

The authors declare no competing interests.

## Supporting information


**Figure S1**. Overview of methylation level and mRNA expression of CDO1 gene in BC tissues from TCGA database. (A) Comparison of CDO1 expression between tumour and normal non‐paired samples in pan‐cancer patients. (B) Pearson correlation analysis between methylation and gene expression. *p* < .05 indicates statistical significance. (C and E) Boxplots showing the ML of cg16707405 and cg07405021 sites between ER‐ and ER+, PR‐ and PR+, HER2‐ and HER2+ BC. (D and F) Boxplots showing the ML of cg16707405 and cg07405021 sites between molecular phenotypes of BC. ML methylation level. Data were presented as means ± SD. ns *p* > .05, *p < .05, **p < .01, ***p < .001, ****p < .0001.

**Figure S2**. The PMR of CDO1 in different clinical features from BC tissues and serums were compared. (A) The PMR of CDO1 in NATs (n = 42), FBA (n = 19) and BC tissues (n = 220) were shown. NATs, normal adjacent tissues. FBA, fibroadenoma. (B‐E) The PMR of CDO1 in T1/2 vs. T3/4, ER− vs. ER+, PR− vs. PR+, and HER2− vs. HER2+ BC patients from tissue were shown. (F) The PMR of CDO1 in Luminal A, Luminal B, HER2‐enriched and basal‐like BC patients from tissues. (G) The PMR of CDO1 in HER2‐enriched and non‐HER2‐enriched BC tissues. (H) The PMR of serum CDO1 in normal people (n = 31), patients with breast benign diseases (n = 37) and BC patients (n = 113) from serum. Data were presented as means ± SD. ns *p* > .05, *p < .05, **p < .01, ***p < .001, ****p < .0001.

**Figure S3. Prediction of transcription factor binding sites (TFBS) in the CDO1 promoter region**. (A) Thirteen transcription factors were predicted in CGI1 region of CDO1 promoter by UCSC Genome Browser on Human (GRCh38/hg38). (B) Seven transcription factors from (A) were identified with specific binding sites within CGI1 region of CDO1 promoter by JASPAR online tool (Figure S3B, Table S14). (C) The correlation between CDO1 expression and transcription factor expression in CDO1 hypomethylated and hypermethylated BC samples was analysed by TCGA methylation data. Statistical significance was set as *p* < .05 in a two‐tailed test. (D) Hypothetical pattern of CDO1 promoter methylation inhibiting CDO1 expression. It possesses three consecutive C2H2‐type zinc‐finger domains at its C‐terminus, enabling it to bind to methylated CpG sites. Additionally, its *N*‐terminus contains the BTB/POZ domain, which facilitates the recruitment of the N‐CoR co‐repressor complex, comprising histone deacetylases. (‐) means that transcription factor inhibits CDO1 expression.

**Figure S4**. Raw Western blotting gels of GAPDH and CDO1 in HepG2, DLD1, MCF‐10A, MCF‐7, MDA‐MB‐231, MDA‐MB‐453 and SK‐BR‐3 cells.

**Figure S5**. Representative figure of methylation sequencing of CDO1 promoter in MCF‐10A cells. Low to high levels of DNA methylation are plotted in yellow‐green‐blue colour‐graded scale (yellow = 0% and blue = 100% methylation).

**Figure S6**. Raw immunoblots of β‐actin, GADPH and CDO1 in BC cells with and without CDO1 overexpression.

**Figure S7**. Raw Western blotting gels of β‐actin and CDO1 in targeted demethylated BC cells.

**Figure S8**. Raw Western blotting gels of β‐actin and CDO1 in targeted demethylation and CDO1 overexpression BC cells in vivo.

**Figure S9**. Raw Western blotting gels of related signalling pathways involved in CDO1.

**Figure S10**. Linear regression correlation analysis of CDO1 promoter PMR in paired tissue and serum.

Supporting Information

**Table S1**. The demographic and clinic‐pathological characteristics of BC tissues, NATs and FBAs of cohort I.

**Table S2**. The demographic and clinic‐pathological characteristics of BC, normal people and benign from serum samples of cohort II.

**Table S3**. Raw data from clinical samples in cohort I and cohort II.

**Table S4**. Primers of CDO1 promoter used for MassARRAY methylation sequencing.

**Table S5**. Primers of CDO1 gene used for MethyLight test.

**Table S6**. Clinical sample information for IHC testing

**Table S7**. Antibodies for Western blot and IHC.

**Table S8**. Primers used for cloning overexpressed‐CDO1 plasmid.

**Table S9**. The sgRNA sequence of targeting CDO1 gene promoter.

**Table S10**. Quantitative real time PCR primers.

**Table S11**. List of ten hypermethylated CpG sites of CDO1 promoter based on TCGA methylation data analysis.

**Table S12**. Description of all effective hypermethylated CpG amplicons studied with the EpiTYPER software.

**Table S13**. Fifteen CpG site information contained in the MethyLight amplified fragment.

**Table S14**. Prediction of transcription factor binding sites (TFBS) in CDO1 promoter region by JASPAR.

**Table S15**. Relationship between the PMR of CDO1 promotor and clinic‐pathologic parameters of 113 BC serums.

**Table S16**. Relationship between CDO1 methylation level combined with HER2 status and metastasis in 113 BC serums.

## Data Availability

Data used in the preparation of this manuscript are available within the article and supplementary data. The RNA‐seq data can be downloaded at: https://www.ncbi.nlm.nih.gov/bioproject/PRJNA933119. All the original raw data have been deposited into the Research Data Deposit public platform (www.researchdata.org.cn), with the approval RDD number as RDDB2023485152. Further information and requests for resources and reagents should be directed to and will be promptly fulfilled by the corresponding authors.
