# Supplementary figures and images for: Targeted demethylation of the CDO1 promoter based on CRISPR system inhibits the malignant potential of breast cancer cells
Source: Clin Transl Med. 2023 Sep 22;13(9):e1423. doi: 10.1002/ctm2.1423 (PMC10517212; doi:10.1002/ctm2.1423)

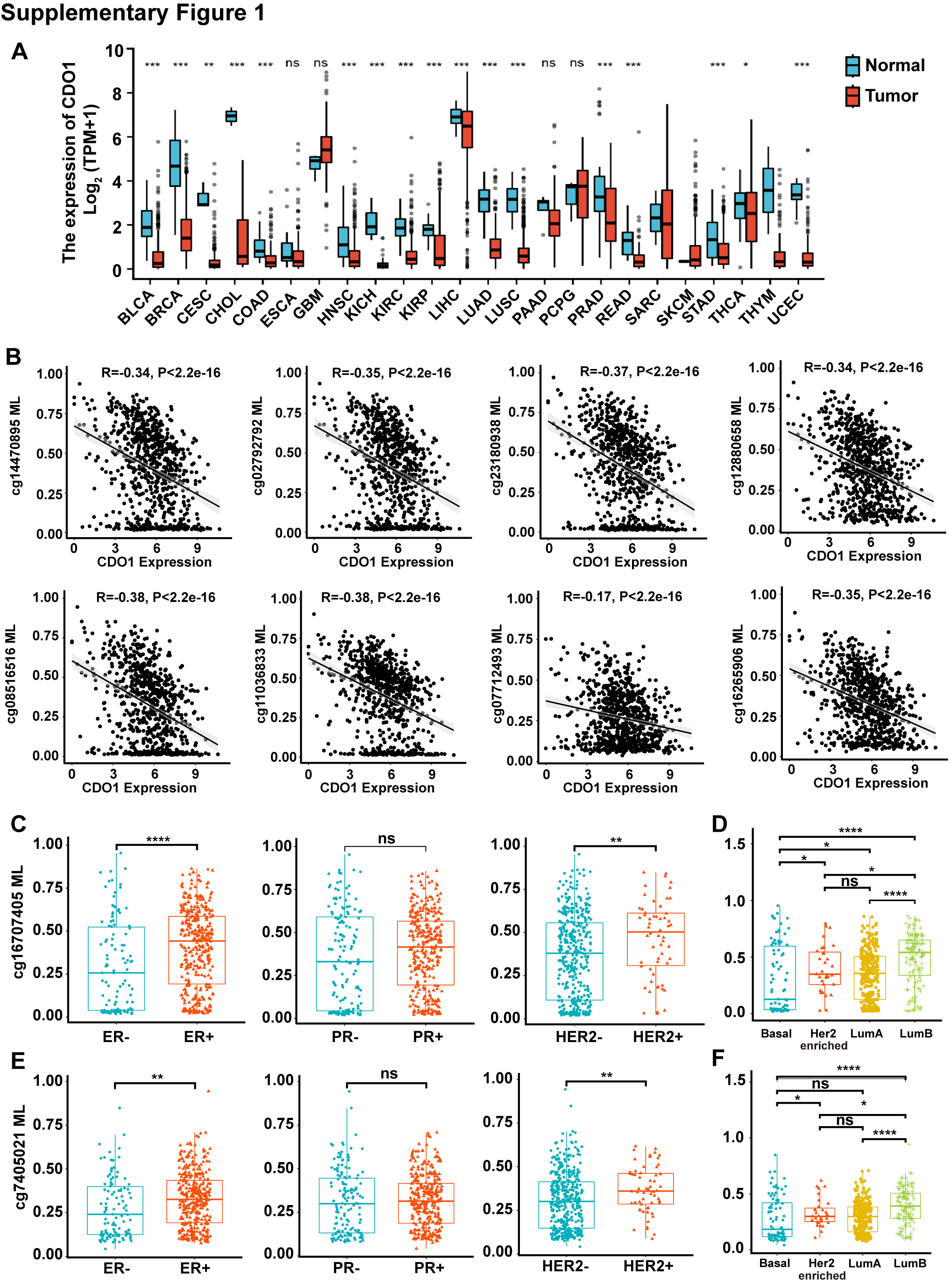

Supplement: Supplementary file 1 — Figure S1. Overview of methylation level and mRNA expression of CDO1 gene in BC tissues from TCGA database. (A) Comparison of CDO1 expression between tumour and normal non‐paired samples in pan‐cancer patients. (B) Pearson correlation analysis between methylation and gene expression. p < .05 indicates statistical significance. (C and E) Boxplots showing the ML of cg16707405 and cg07405021 sites between ER‐ and ER+, PR‐ and PR+, HER2‐ and HER2+ BC. (D and F) Boxplots showing the ML of cg16707405 and cg07405021 sites between molecular phenotypes of BC. ML methylation level. Data were presented as means ± SD. ns p > .05, *p < .05, **p < .01, ***p < .001, ****p < .0001. [file CTM2-13-e1423-s022.tif]

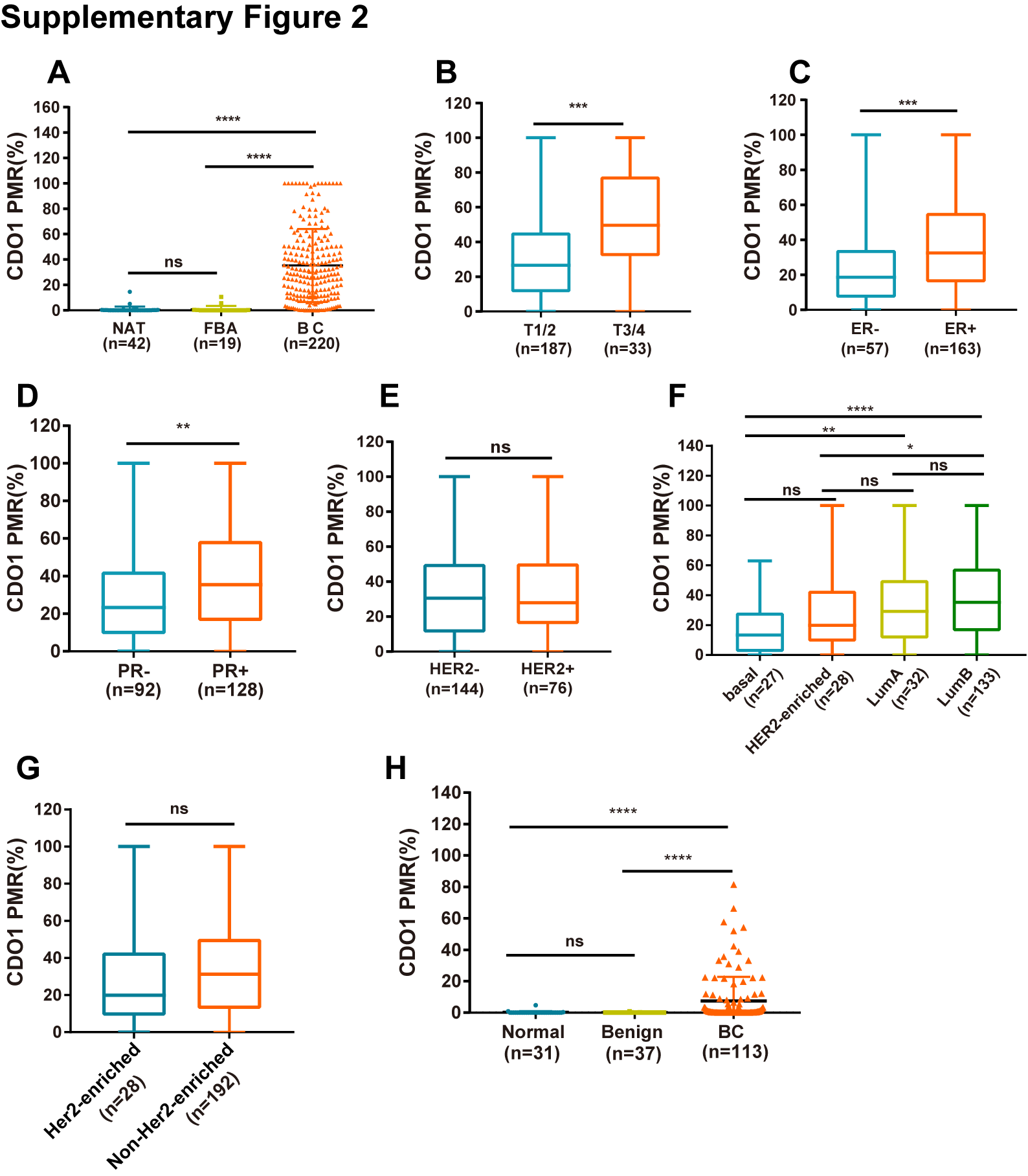

Supplement: Supplementary file 2 — Figure S2. The PMR of CDO1 in different clinical features from BC tissues and serums were compared. (A) The PMR of CDO1 in NATs (n = 42), FBA (n = 19) and BC tissues (n = 220) were shown. NATs, normal adjacent tissues. FBA, fibroadenoma. (B‐E) The PMR of CDO1 in T1/2 vs. T3/4, ER− vs. ER+, PR− vs. PR+, and HER2− vs. HER2+ BC patients from tissue were shown. (F) The PMR of CDO1 in Luminal A, Luminal B, HER2‐enriched and basal‐like BC patients from tissues. (G) The PMR of CDO1 in HER2‐enriched and non‐HER2‐enriched BC tissues. (H) The PMR of serum CDO1 in normal people (n = 31), patients with breast benign diseases (n = 37) and BC patients (n = 113) from serum. Data were presented as means ± SD. ns p > .05, *p < .05, **p < .01, ***p < .001, ****p < .0001. [file CTM2-13-e1423-s024.tif]

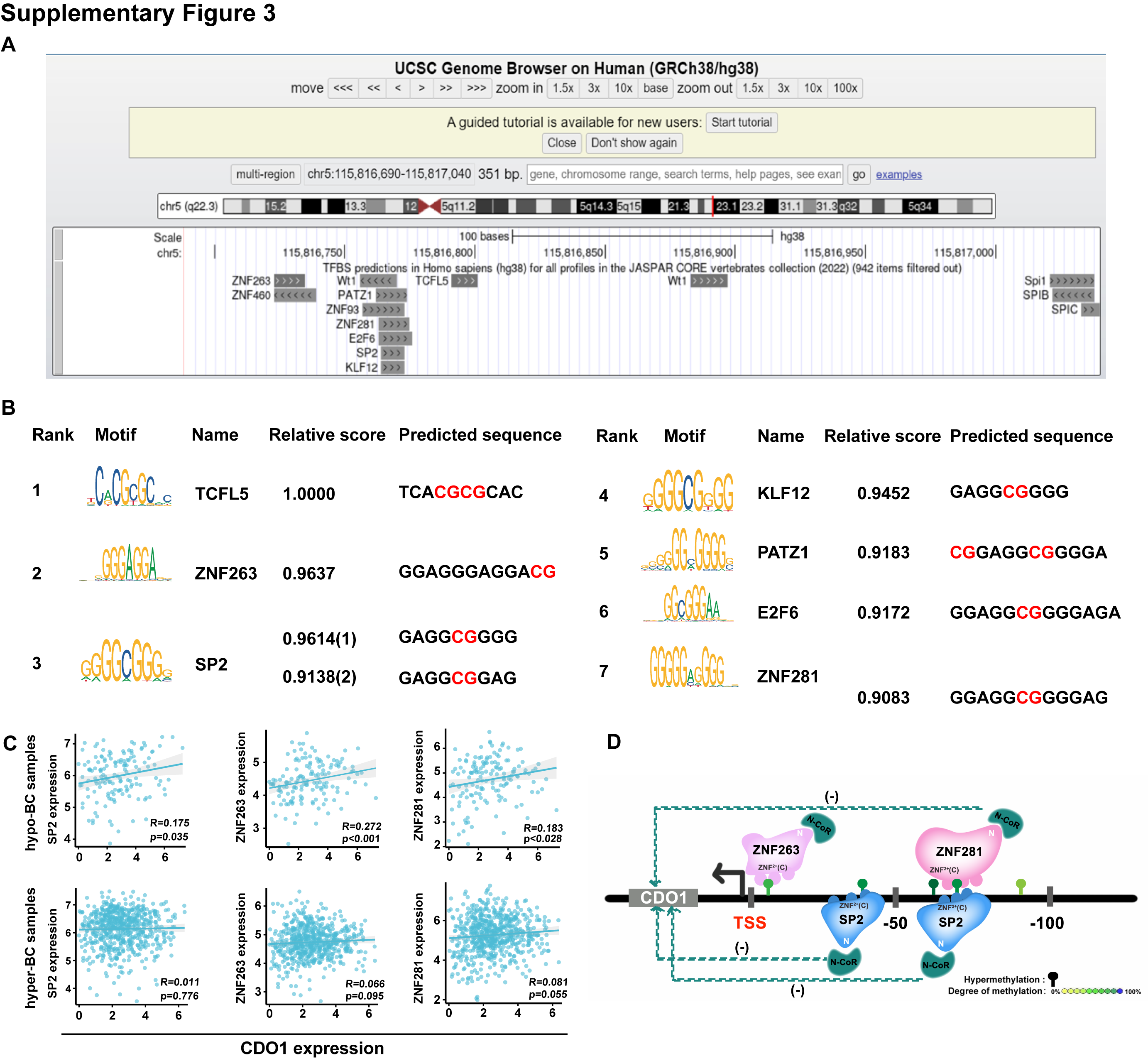

Supplement: Supplementary file 3 — Figure S3. Prediction of transcription factor binding sites (TFBS) in the CDO1 promoter region. (A) Thirteen transcription factors were predicted in CGI1 region of CDO1 promoter by UCSC Genome Browser on Human (GRCh38/hg38). (B) Seven transcription factors from (A) were identified with specific binding sites within CGI1 region of CDO1 promoter by JASPAR online tool (Figure S3B, Table S14). (C) The correlation between CDO1 expression and transcription factor expression in CDO1 hypomethylated and hypermethylated BC samples was analysed by TCGA methylation data. Statistical significance was set as p < .05 in a two‐tailed test. (D) Hypothetical pattern of CDO1 promoter methylation inhibiting CDO1 expression. It possesses three consecutive C2H2‐type zinc‐finger domains at its C‐terminus, enabling it to bind to methylated CpG sites. Additionally, its N‐terminus contains the BTB/POZ domain, which facilitates the recruitment of the N‐CoR co‐repressor complex, comprising histone deacetylases. (‐) means that transcription factor inhibits CDO1 expression. [file CTM2-13-e1423-s007.tif]

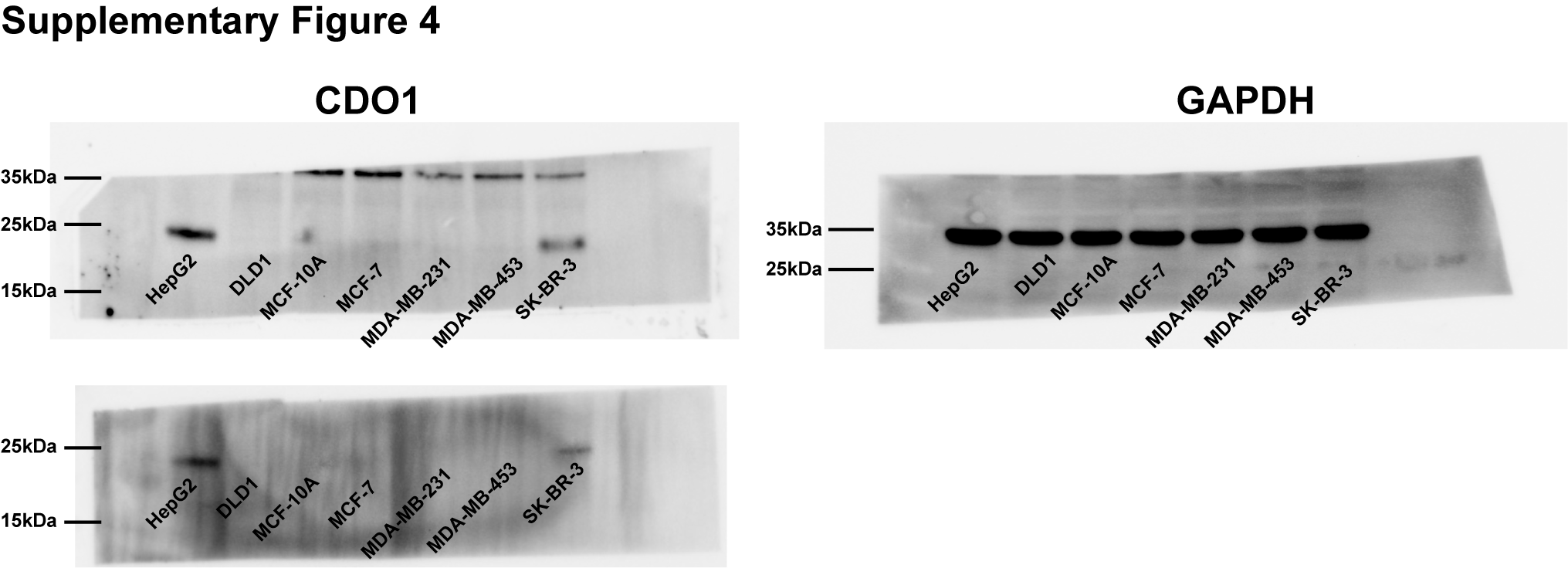

Supplement: Supplementary file 4 — Figure S4. Raw Western blotting gels of GAPDH and CDO1 in HepG2, DLD1, MCF‐10A, MCF‐7, MDA‐MB‐231, MDA‐MB‐453 and SK‐BR‐3 cells. [file CTM2-13-e1423-s018.tif]

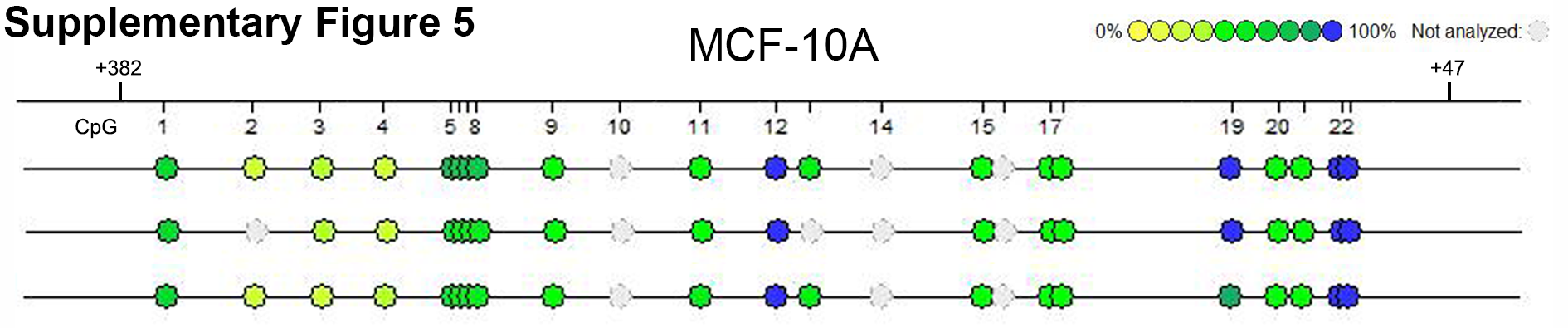

Supplement: Supplementary file 5 — Figure S5. Representative figure of methylation sequencing of CDO1 promoter in MCF‐10A cells. Low to high levels of DNA methylation are plotted in yellow‐green‐blue colour‐graded scale (yellow = 0% and blue = 100% methylation). [file CTM2-13-e1423-s003.tif]

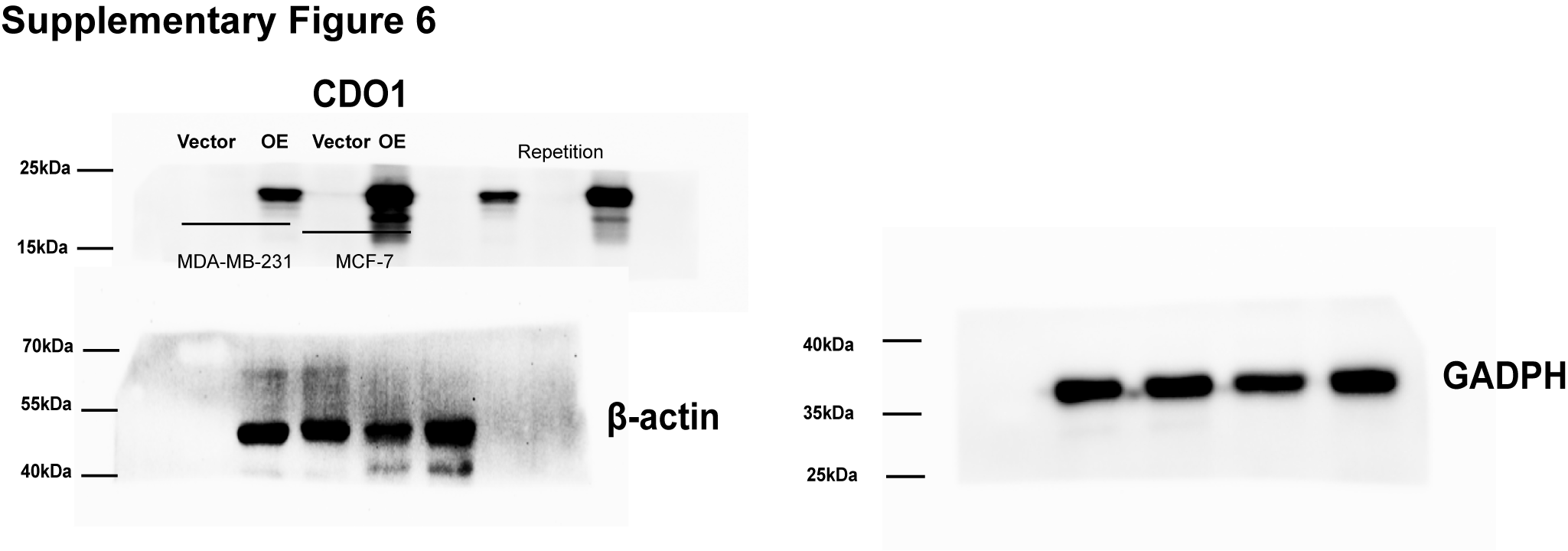

Supplement: Supplementary file 6 — Figure S6. Raw immunoblots of β‐actin, GADPH and CDO1 in BC cells with and without CDO1 overexpression. [file CTM2-13-e1423-s027.tif]

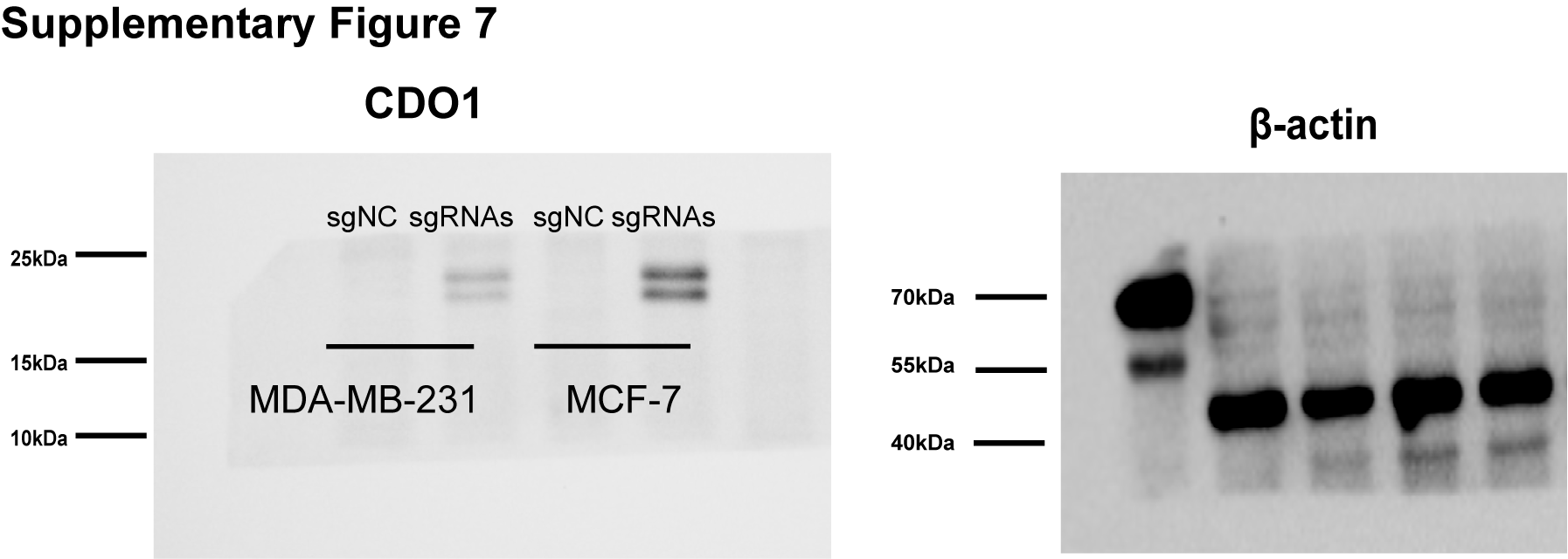

Supplement: Supplementary file 7 — Figure S7. Raw Western blotting gels of β‐actin and CDO1 in targeted demethylated BC cells. [file CTM2-13-e1423-s020.tif]

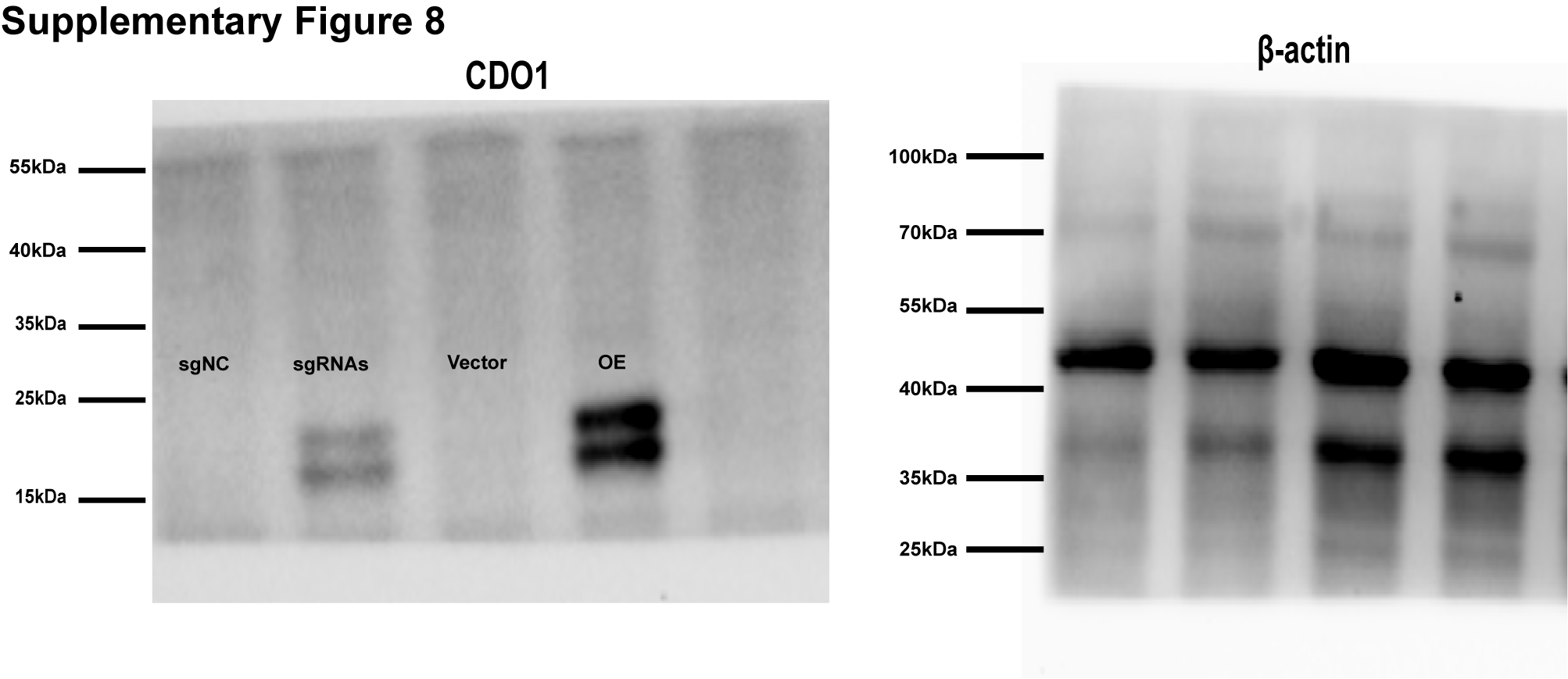

Supplement: Supplementary file 8 — Figure S8. Raw Western blotting gels of β‐actin and CDO1 in targeted demethylation and CDO1 overexpression BC cells in vivo. [file CTM2-13-e1423-s014.tif]

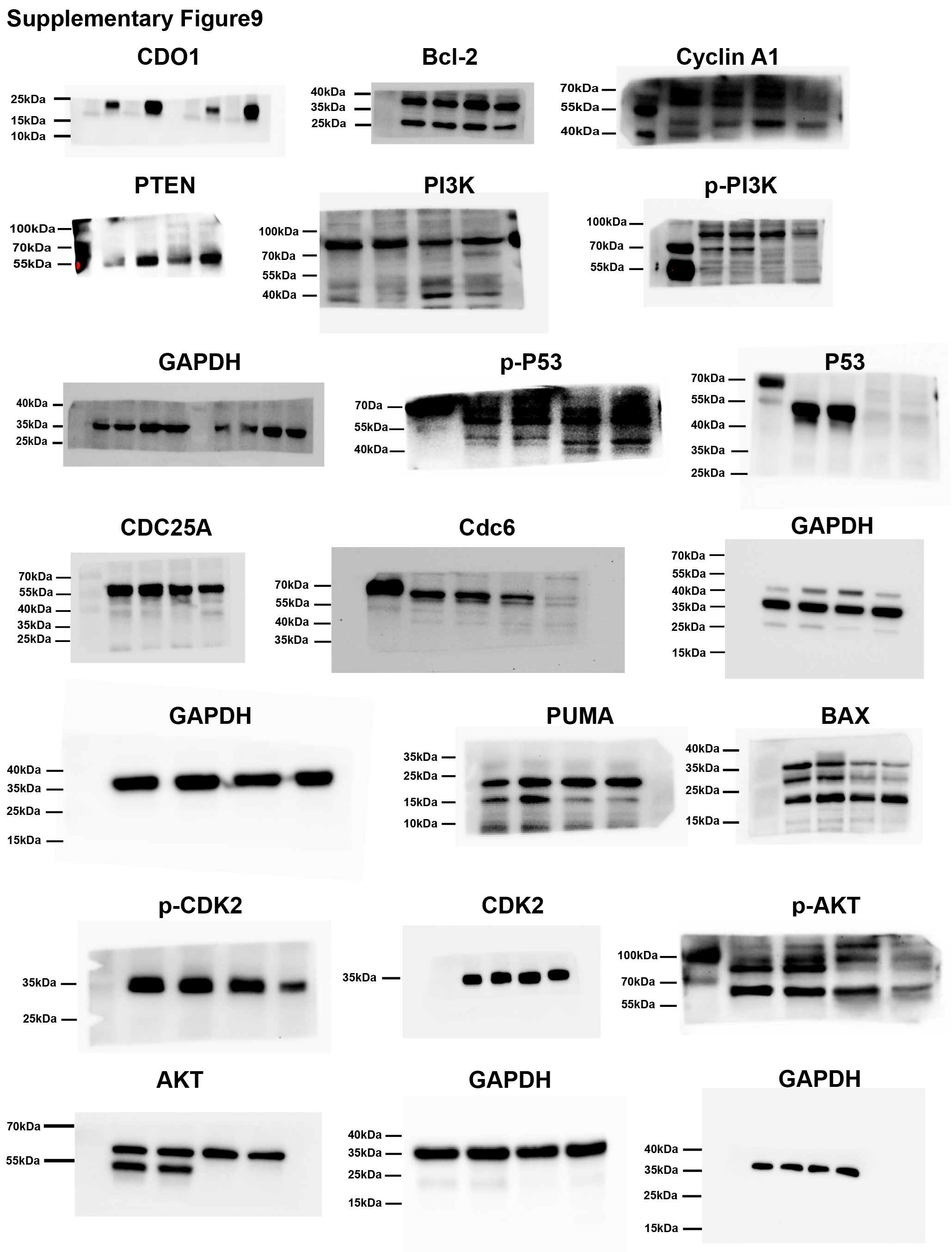

Supplement: Supplementary file 9 — Figure S9. Raw Western blotting gels of related signalling pathways involved in CDO1. [file CTM2-13-e1423-s023.tif]

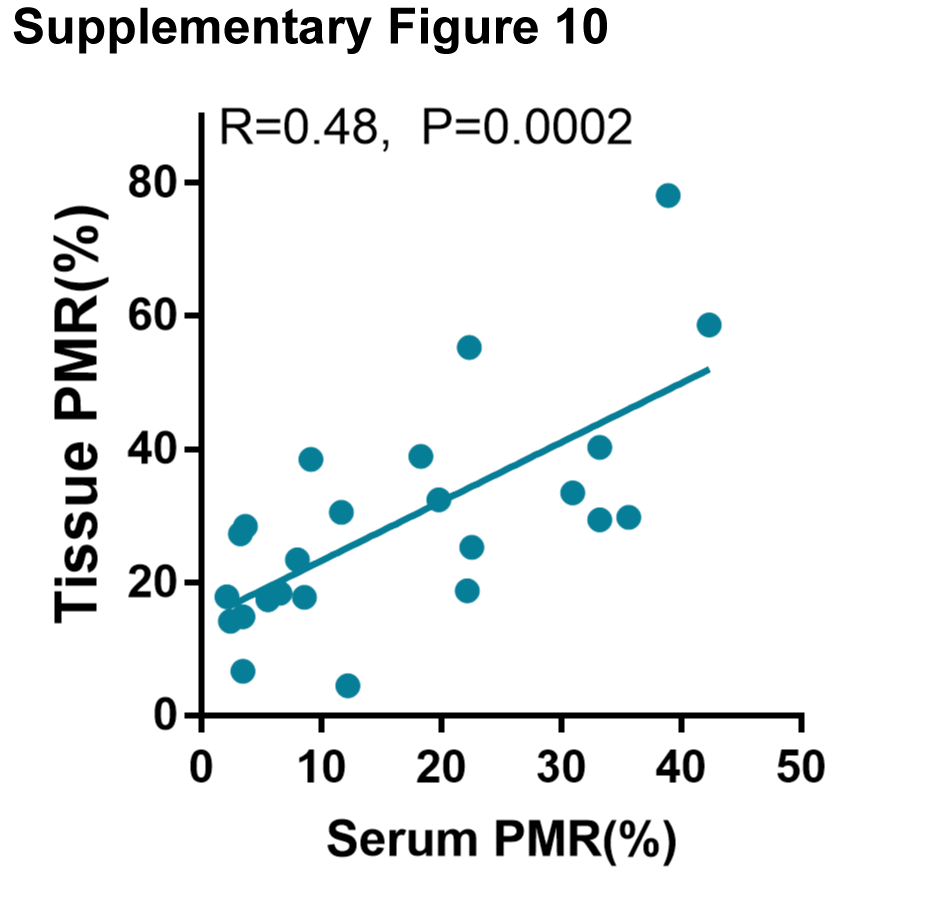

Supplement: Supplementary file 10 — Figure S10. Linear regression correlation analysis of CDO1 promoter PMR in paired tissue and serum. [file CTM2-13-e1423-s009.tif]
