# Supplementary material for: Targeted demethylation of the CDO1 promoter based on CRISPR system inhibits the malignant potential of breast cancer cells
Source: Clin Transl Med. 2023 Sep 22;13(9):e1423. doi: 10.1002/ctm2.1423 (PMC10517212; doi:10.1002/ctm2.1423)
Supplement: Supplementary file 11 — Supporting Information [file CTM2-13-e1423-s011.docx]

**SUPPLEMENTARY** **MATERIALS AND METHODS**

1. ***DNA Isolation and Bisulfite Conversion***

Total DNA was isolated from tissues using the TIANampFFPE DNA kit (TIANGEN, Beijing, China). cfDNA was extracted from 1 mL serum using the QIAamp DNA Blood Mini Kit (Qiagen, Dusseldorf, Germany). The QIAamp DNA Mini Kit (Qiagen, Dusseldorf, Germany) was used to extract DNA from fresh tissues and cell lines. DNA bisulfite conversion was performed using the EZ DNA Methylation Kit (Zymo Research, Orange County, CA, USA), and total of 20 µL modified DNA was obtained, which could be used immediately for the next experiment or stored at -80 °C for future use.

1. ***DNA methylation*** ***sequencing analysis***

Fourteen BC samples and 17 NATs samples from cohort I were used for DNA methylation sequencing analysis. The target-specific primer pairs were designed using the EpiDesigner software. The reverse primer had a T7 promoter tag for transcription (5′-CAGTAATACGACTCACTATAGGGAGAAGGCT-3′) and the forward primer was tagged with a 10-mer (5′-AGGAAGAGAG-3′) to balance the melting temperature^1^. The CDO1 promoter methylation sequencing generates a 351 bp PCR fragment (+18 to -332). Primers were synthesized by RUIBIO (Guangzhou, China). The PCR-amplified products were treated with shrimp alkaline phosphatase, and in vitro transcription and base-specific cleavage were performed simultaneously^1^. With base-specific cleavage, these changes appear as G or A changes in the cleavage products produced by the posterior strand, resulting in a mass difference of 16 Daltons (Da) per CpG site, as determined by MassARRAY mass spectrometry^2^. Mass spectrometry can be used to calculate the relative degree of methylation by comparing the signal intensity between methylated and unmethylated DNA mass signals.

Since DNA fragments of different masses are required, DNA fragments of the same size or outside the mass range of 1700-7000 Da cannot be detected, and therefore methylation data are not provided^2^. For example, when using EpiTYPER (Agena Bioscience, San Diego, CA, USA) to analyze results, the CpG Units data errors of L-mass/H-mass, D, OL and SN/SM appeared in the “Comment” column were excluded because its data was too large to be used. EpiTYPER detects methylation levels in appropriately sized DNA fragments as CpG sets that contain one or more CpG sites (typically 2-4 CpGs) within the same cut fragment. Thus, multiple CpG sites within a cluster are recognized when they are close to each other. In this study, a total of twenty-three amplicons containing thirty-seven CpG sites were analyzed with EpiTYPER software. In this article, we used the term “CpG site” regardless of whether one CpG was detected or clusters of CpGs are present within a single short DNA fragment.

1. ***MethyLight assay***

MethyLight is a fluorescent quantitative PCR based on TaqMan, which depends on the hybridization and cleavage of probes targeting the CpG of interest^29^. Bisulfite-converted DNA (Bis-DNA) was used as a template for MethyLight assay. A total of 25 μl reaction contains 2 μl Bis-DNA, 300nmol/l each primer, 200nmol/l fluorescent probe, and 10μl 2 x Methylation PCR Master Mix. Reactions was done at 95゜C for 5min followed by 15 cycles at 95゜C for 15 sec, 64゜C for 30 sec and 30 cycles at 95゜C for 15 sec, 62゜C for 32 sec to collect fluorescence. The mean cycle threshold (Ct) value of each sample after repeated detection was used for analysis.

We generated the positive control of CDO1 promoter methylation as following. DNA from normal breast tissue was treated with the M.SssI methyltransferase (New England Biolabs, Ipswich, MA) in vitro to make all CG sites on sequence methylated. PMR value defines the percentage of methylated molecules at a specific locus and was calculated as the ratio of the methylation of a gene of interest normalized to each control gene relative to that of the M.SssI-treated sample^3^. The PMR was calculated using the formula:

| PMR = 2^−ΔΔCt^ × 100% | (1) |
| --- | --- |
| Where −ΔΔCt = ΔCt_sample_ - ΔCt_control_ = [Ct_CDO1_- Ct_ACTB_]_sample_ - [Ct_CDO1_- Ct_ACTB_]_positive control (M.SssI-treated sample)_. |  |

Based on Youden's index, the optimal cut-off was determined. When the PMR value exceeded the cut-off value, the samples were classified as methylation positive (methylation). Unmethylated samples were defined as those with PMR values below the cut-off value.

1. ***Cell culture and chemicals***

Human breast cancer cell lines (MCF-7, MDA-MB-231, MDA-MB-453 and SK-BR-3), HEK-293T and human normal mammary epithelial cell line (MCF-10A) were purchased from Guangzhou Suyan Biotechnology Co., Ltd. Human hepatocarcinoma cell line (HepG2) and human colorectal cancer epithelial cell line (DLD1) were provided by Dr. Yi Zhou (Guangzhou). Cell lines were grown in 5% CO2 humidified incubators at 37°C with penicillin and streptomycin supplemented with 100 units in each ml (Thermo Fisher, Waltham, Massachusetts) and 10% FBS. In addition to the RSL3, ferrostatin-1 (Fer-1), and 5-aza-2-deoxycytidine (5-Aza), we purchased them from Selleck and Sigma-Aldrich, respectively.

1. ***Immunohistochemistry (IHC)***

The expression of CDO1 protein in BC tissues and NATs was detected using immunohistochemical EnVision two-step method^4^. Tissue sections were deparaffinized, rehydrated, and antigen retrieved. Afterwards, the sections were incubated overnight at 4°C with primary antibodies against CDO1. Incubation of the second antibody at 37°C for 25 minutes had been carried out. Diaminobenzidine (DAB) chromogenic agent was used for visualization. AxioScan.Z1 (Carl Zeiss AG, Oberkochen, Germany) was used to scan all slides and HALO Image Analysis Platform (Indica Labs, Albuquerque, New Mexico, USA) was used to quantify CDO1 expression with "cytoplasmic IHC quantification" module, and H-score of each case was calculated.

1. ***Quantitative real time PCR analysis (qRT-PCR)***

TRIzol reagent (Invitrogen, Carlsbad, CA, USA) was used to extract total RNA from cell lines and tissues. 1 µg total RNA was reverse transcribed into cDNA using Prime Script RT reagent Kit (TAKARA, Kyoto, Japan). In this study, qRT-PCR was performed with TB Green Premix EX Taq kits (TAKARA, Kyoto, Japan) on a Bio-Rad CFX96 Touch Real-Time instrument (Bio-Rad, CA, USA). The samples were analyzed three times. The expression of each gene was expressed as 2*^−ΔΔCt^* and normalized to the housekeeping gene *ACTB*.

1. ***Western blot analysis (WB)***

Proteins were extracted from tissue and cell lines using the Whole Cell Lysis Assay kit (KeyGEN, Nanjing, China). Using a BCA (bicinchoninic acid) protein assay kit (Beyotime, Shanghai, China), we measured protein concentrations. Equal amounts of proteins, depending on the molecular sizes, were separated by 4-13% SDS-PAGE, transferred to nitrocellulose membranes (Eck Millipore, Darmstadt, Germany) and blocked with TBST containing 5% bovine serum albumin. ImageJ was used to quantify protein levels, and relative expression of all target genes was normalized to GAPDH, a housekeeping gene.

1. ***CCK-8 assay, colony-forming assay and transwell assay***

The cells were respectively seeded in 96-well culture plates (1× 10^4^/well) and cultured for five days. After the end of culture, 10 μL CCK8 reagent (DOJINDO, Shanghai, China) was added to each experimental well and then incubate at 37 °C for 2 h. The absorbance of each experimental well at 450 nm was measured using a microplate reader (BioTek Epoch, Inc., Winooski, VT, USA).

Cells of 1 × 10^3^ were seeded into the six-well plate for colony formation. After 13 days of cultivation, colonies were fixed with 4% paraformaldehyde at room temperature and stained with 1% crystal violet for 30 minutes. We counted colonies with more than 100 cells with a microscope (Leica Microsystems, Wetzlar, Germany).

Transwell assay was used to measure cell invasion and migration. Transwell chambers were loaded with or without pre-coated Matrigel membranes into 24-well plates. The upper chamber contained 200 µL of serum-free medium with 1.0×10^5^ cells/ml, and the lower chamber contained 600 µL of medium with 15% FBS. Chambers were removed at the stipulated times, and penetrating cells were fixed for 30 min in 4% paraformaldehyde, then dyed for 20 min with 0.1% crystal violet. We counted penetration cells in four randomly selected fields of each sample (magnification 20 ×). Matrigel was pre-coated on the transwell chambers for invasion assays.

1. ***In vivo animal experiment***

The right scapular region of female BALB/c nude mice (6-week-old) was injected with 1 × 10^6^ cells per 50μL PBS plus 50μL Matrigel (n = 5 per group). Every four days, mice were weighed and tumors were measured. Mice tumors were weighted and collected at the 32th day. Tumor volume (V) was measured by vernier caliper and calculated by the standard formula: *V=L×W^2^/2*. L for length and W for width. For the metastatic model, the 6×10^5^ cells BC cells (in 200 µl PBS) were injected into the tail vein of each mouse (n = 5/ group). For the purpose of detecting metastasis in the lungs, hematoxylin and eosin (HE) staining was carried out after 40 days. The tumor tissues were then examined by MethyLight, RT-PCR and WB.

1. ***RNA sequencing (RNA-seq)***

RNA integrity was analyzed with Agilent 2100 Bioanalyzer (Agilent Technologies, Santa Clara, CA, USA). RNA concentration was tested by Qubit RNA Assay Kit in Qubit Fluorometer (Invitrogen, Carlsbad, CA, USA). Total RNA samples that meet the following requirements were used in subsequent experiments: RNA integrity number (RIN) ≥ 7.0 and a 28S:18S ratio ≥ 1.5. RNA sequencing was performed on an Illumina platform to select differentially expressed genes (DEGs) between Vector and CDO1 overexpressed BC cell lines. The Kyoto Gene and Genome Encyclopedia (KEGG) and gene ontology (GO) analysis were used to evaluate the DEGs. Sequencing libraries were generated and sequenced by CapitalBio Technology (Beijing, China). Fastq files were generated via Illumina bcl2fastq2 from and bcl files were produced by an Illumina NextSeq sequencer. The quality of individual sequences was evaluated using FastQC software after adapter trimming with Cutadapt software. The human genome reference used for alignment was hg38. Transcript abundance was measured in fragments per kb of exon per million fragments mapped (FPKM).

1. ***Cell cycle analysis***

Cell Cycle Kit (KeyGEN, Nanjing, China) was used to detect distribution of cell cycle phages. First, cells (1×10^6^) were digested with trypsin and then washed twice with PBS. Next, the cells were fixed overnight with 500 μL 70% cold ethanol. After washing off the fixed solution with PBS, the cells were stained with 500 μL staining working solution (Rnase A:PI=1:9) and incubated for 60 min at 25 ℃. CytoFLEX S flow cytometer (Beckman Coulter, Inc.) was used to measure the red fluorescence of 10,000 cells at 488nm, and then CytExpert 2.3 (Beckman Coulter, Inc.) was used to analyze the results^5^.

1. ***Cell apoptosis analysis***

Cell apoptosis was detected using Annexin V-APC/PI Apoptosis Detection Kit (KeyGEN, Nanjing, China). First, cells (5×10^5^) were digested with trypsin without EDTA, and then washed twice with PBS. After the cells were resuscitated with 500 μL Binding Buffer, 5 μL PI and 5 μL Annexin V-APC dye solution were added. CytoFLEX S flow cytometer (Beckman Coulter, Inc.) was used to record the fluorescence signal for 10,000 cells, which was then analyzed using CytExpert 2.3. Early apoptotic cell showed PI‑negative and Annexin V‑APC‑positive; late apoptotic cell showed PI‑positive and Annexin V‑APC‑positive.

1. ***Measurement of lipid ROS, and intracellular iron levels***

Cells (1×10^6^) were collected in the culture medium containing 10 μM C11-BODIPY and 10% FBS and then incubated at 37 ℃ from light for 30 min. Then, the cells were washed twice with PBS and 10,000 cells were recorded by flow cytometry. An argon laser (excitation wavelength 488 nm; emission, emission wavelength 500-535 nm) was used to oxidize C11 BODIPY, whereas a white light laser (excitation wavelength 561 nm; emission spectrum, 573-613 nm) was used for unoxidized C11 BODIPY.

Cells (2×10^6^) were rapidly homogenized in 5 volumes of iron assay buffer to release iron. To measure total iron, 5 μL iron reducer was added to each of the sample wells in a 96 well plate to reduce Fe^3+^ to Fe^2+^. To measure ferrous iron, 5 μL iron assay buffer was added to each of the sample wells. The reaction solution was shaken and incubated at room temperature in the dark for 30 min. Then, 100 μL iron probe was added to each well and the reaction was incubated at room temperature from light for 60 min after shaking. Finally, the absorbance was measured at 593 nm.

**REFERENCES**

**1.** Wu N, Yuan F, Yue S, et al. Effect of exercise and diet intervention in NAFLD and NASH via GAB2 methylation. *Cell & bioscience*. 2021;11(1):189.

**2.** Freire-Aradas A, Phillips C, Mosquera-Miguel A, et al. Development of a methylation marker set for forensic age estimation using analysis of public methylation data and the Agena Bioscience EpiTYPER system. *Forensic science international. Genetics*. 2016;24:65-74.

**3.** Singh A, Gupta S, Badarukhiya JA, Sachan M. Detection of aberrant methylation of HOXA9 and HIC1 through multiplex MethyLight assay in serum DNA for the early detection of epithelial ovarian cancer. *International journal of cancer*. 2020;147(6):1740-1752.

**4.** Zhang YH, Cheng YH, Cai G, Zhang YJ. Expression and significance of aquaporin-4 in thyroid carcinoma. *Multiple sclerosis and related disorders*. 2021;48:102726.

**5.** Ke M, Lin F, Wang H, et al. Sigma‑1 receptor overexpression promotes proliferation and ameliorates cell apoptosis in β‑cells. *Molecular medicine reports*. 2022;25(5).
